# The Soil Microbiome of GLORIA Mountain Summits in the Swiss Alps

**DOI:** 10.3389/fmicb.2019.01080

**Published:** 2019-05-15

**Authors:** Magdalene Adamczyk, Frank Hagedorn, Sonja Wipf, Jonathan Donhauser, Pascal Vittoz, Christian Rixen, Aline Frossard, Jean-Paul Theurillat, Beat Frey

**Affiliations:** ^1^Forest Soils and Biogeochemistry, Swiss Federal Institute for Forest, Snow and Landscape Research WSL, Birmensdorf, Switzerland; ^2^Community Ecology, WSL Institute for Snow and Avalanche Research SLF, Davos, Switzerland; ^3^Institute of Earth Surface Dynamics, University of Lausanne, Lausanne, Switzerland; ^4^Fondation J.-M. Aubert, Champex-Lac, Switzerland; ^5^Department of Botany and Plant Biology, University of Geneva, Chambésy, Switzerland

**Keywords:** alpine, bacteria, fungi, climate change, mountain summit, soil, GLORIA

## Abstract

While vegetation has intensively been surveyed on mountain summits, limited knowledge exists about the diversity and community structure of soil biota. Here, we study how climatic variables, vegetation, parent material, soil properties, and slope aspect affect the soil microbiome on 10 GLORIA (Global Observation Research Initiative in Alpine environments) mountain summits ranging from the lower alpine to the nival zone in Switzerland. At these summits we sampled soils from all four aspects and examined how the bacterial and fungal communities vary by using Illumina MiSeq sequencing. We found that mountain summit soils contain highly diverse microbial communities with a total of 10,406 bacterial and 6,291 fungal taxa. Bacterial α-diversity increased with increasing soil pH and decreased with increasing elevation, whereas fungal α-diversity did not change significantly. Soil pH was the strongest predictor for microbial β-diversity. Bacterial and fungal community structures exhibited a significant positive relationship with plant communities, indicating that summits with a more distinct plant composition also revealed more distinct microbial communities. The influence of elevation was stronger than aspect on the soil microbiome. Several microbial taxa responded to elevation and soil pH. *Chloroflexi* and *Mucoromycota* were significantly more abundant on summits at higher elevations, whereas the relative abundance of *Basidiomycota* and *Agaricomycetes* decreased with elevation. Most bacterial OTUs belonging to the phylum *Acidobacteria* were indicators for siliceous parent material and several OTUs belonging to the phylum *Planctomycetes* were associated with calcareous soils. The trends for fungi were less clear. Indicator OTUs belonging to the genera *Mortierella* and *Naganishia* showed a mixed response to parent material, demonstrating their ubiquitous and opportunistic behaviour in soils. Overall, fungal communities responded weakly to abiotic and biotic factors. In contrast, bacterial communities were strongly influenced by environmental changes suggesting they will be strongly affected by future climate change and associated temperature increase and an upward migration of vegetation. Our results provide the first insights into the soil microbiome of mountain summits in the European Alps that are shaped as a result of highly variable local environmental conditions and may help to predict responses of the soil biota to global climate change.

## Introduction

Average global surface temperatures are projected to rise in the range of 1.1 to 6.4°C by the end of the 21^st^ century compared to pre-industrial levels (CH2018, 2018) and changes in temperature are known to be more pronounced at higher elevations (Pepin et al., 2015). Mountain plant communities provide important early indicators to changing climate (Körner, 2003; Rixen and Wipf, 2017; Steinbauer et al., 2018). In the European Alps, repeated investigations have indicated upward expansions of alpine grassland species and an increase in species richness at higher elevations over the past decades (Gottfried et al., 2012; Pauli et al., 2012; Matteodo et al., 2013; Wipf et al., 2013; Rumpf et al., 2018; Steinbauer et al., 2018).

The alpine soil microbiome plays key roles in the processes of weathering, pedogenesis, biogeochemical cycling, and plant colonisation of bare soils, and thus crucially shapes the nutrient cycling of alpine ecosystems (Nemergut et al., 2007; Frey et al., 2010; Margesin, 2012; Donhauser and Frey, 2018). Despite harsh environmental conditions alpine soils harbour a considerable microbial diversity (Rime et al., 2015; Frey et al., 2016; Malard and Pearce, 2018). Mountainous terrain, and especially mountain summits exhibit a large variability in biotic and abiotic conditions (Scherrer and Körner, 2011; Winkler et al., 2016; Kulonen et al., 2018) that offer a unique opportunity to study how soil organisms respond to the variability in both climate and vegetation. Due to climate change, alterations of vegetation composition and plant litter quality at higher altitudes may induce changes in the diversity and composition of microbial decomposers (Rudgers et al., 2014; Donhauser and Frey, 2018; Matteodo et al., 2018).

Elevational gradients have been used as proxies for the impacts of climate change on above and belowground organisms including plants and soil microbes in cold biomes (Donhauser and Frey, 2018). While plant diversity decreases with increasing elevation (Theurillat et al., 2003; Vittoz et al., 2010; Pauli et al., 2012), soil microbial communities appear to be more versatile and adapted to grow in these inhospitable, cold habitats (Margesin and Miteva, 2011; Zumsteg et al., 2012). Numerous studies have found that bacterial taxon richness decreases along alpine elevational gradients with distinct shifts of climatic conditions (Bryant et al., 2008; Singh et al., 2013, 2014; Hofmann et al., 2016), however, a study by Wu et al. (2017) did not show a decline of bacterial taxon richness along such a gradient in alpine soils of Mount Cardrona, New Zealand. Elevational distribution patterns have also been reported for soil fungal communities, albeit with no universal trend (Nottingham et al., 2016; Ni et al., 2018). Ni et al. (2018), for example, found a linear increase in fungal diversity along an elevation gradient of 2000 to 2500 m a.s.l. in the alpine tundra on the Changbai mountain in China, yet other studies found the opposite pattern (Matsuoka et al., 2016; Nottingham et al., 2016; Tian et al., 2017). Further research has suggested that fungal diversity and richness have no clear relationships with elevation (Coince et al., 2014) or that fungal composition, but not richness, varies across elevational gradients (Shen et al., 2014; Lanzén et al., 2016). These controversial findings document a lack of universal patterns and that elevation in itself is not the main driver shaping bacterial and fungal communities in alpine soils (Pellissier et al., 2014; Yashiro et al., 2016).

Elevation-dependent effects on composition of microbial communities across mountain environments can further be confounded by parent material in alpine ecosystems (Lazzaro et al., 2009; Larouche et al., 2012; Shen et al., 2013; Reith et al., 2015). In addition, slope aspect and exposition (Zumsteg et al., 2013; Liu L. et al., 2015; Frey et al., 2016; Wu et al., 2017; Chai et al., 2018) can modify elevational patterns as soil temperatures can vary considerably over distances of only a few metres (Scherrer and Körner, 2011). These studies suggest that factors other than elevation alone, such as soil properties, temperature, topography and vegetation influence the alpine soil microbiome (Lanzén et al., 2015, 2016; Siles and Margesin, 2016). Overall, there is a considerable lack of knowledge about the environmental drivers affecting the high-alpine soil microbiome, in particular, on mountain summits. Understanding these drivers, however, is crucial to predict the impact of upward shifts of plant species in response to climatic warming on the soil microbial communities in alpine environments.

Summits in mountain ecosystems are well-suited for climate studies on both plant diversity and soil microbial communities due to the minimal confounding effects present, such as a lack of input from transported rock material by landslides (Körner, 2007, 2016; Steinbauer et al., 2018). Repeated vegetation surveys on mountain summits have documented an upward shift of plant species in response to climatic warming (Steinbauer et al., 2018). This shift has likely also affected the root-associated soil microbiome, which is often closely functionally linked to alpine plants (Peay et al., 2013; Yashiro et al., 2016). Increasing temperature could therefore change the diversity and composition of the soil microbiome either directly or indirectly via the colonisation by new, more thermophilous species and lead to an increase of plant cover. While numerous vegetation surveys have been carried out on mountain summits, comparisons of soil bacterial and fungal communities on mountain summits at different elevations and aspects in the European Alps have, to our knowledge, not yet been conducted. Knowing the current diversity patterns and community structures of functionally and taxonomically strongly differing organism groups will be fundamental to elucidating potential responses to ongoing climatic changes on mountain summits.

In this article, we studied the soil bacterial and fungal distribution patterns at 10 different mountain summits of the Global Observation Research Initiative in Alpine environments (GLORIA) and explored the impact of parent material, elevation, slope aspect, soil properties, and vegetation on the soil microbiome. The 10 summits ranging from alpine grassland near the treeline ecotone (2360 m a.s.l.) across scarcely vegetated areas to the virtually bare nival biome (3212 m a.s.l.) are situated in the Swiss National Park and the canton of Valais on two types of parent material. Plant richness and composition together with soil properties were measured simultaneously at the same summits to enable a direct comparison between bacterial, fungal and plant community patterns. We tested the hypotheses (i) that microbial α-diversity declines with increasing elevation; (ii) that bacterial and fungal community structures change with slope aspect, since soil temperature, vegetation and soil properties vary with aspect; and (iii) that variation in bacterial and fungal community structures correlates predominantly with soil pH, as parent material strongly modifies the distribution patterns either directly or indirectly by altered soil properties and vegetation. Our results provide fundamental and novel insights into the unexplored soil microbiome of mountain summits in the European Alps. As these summits are currently experiencing significant temperature increases and upward migrations of plants, this study may help to predict responses of the belowground community to such environmental changes as forecasted global temperatures continue to rise.

## Materials and Methods

### Field Sites and Soil Collection

Ten alpine mountain summits of the three Swiss target regions of the Global Observation Research Initiative in Alpine environments (GLORIA) were selected for this study. GLORIA is a world-wide long-term monitoring network, which was initiated to gain knowledge about the impact of climate warming on mountain ecosystems (Pauli et al., 2015). The target regions SN1 and SN2 are located in the region of the Swiss National Park in Grisons in south-eastern Switzerland, whereas the third target region VAL is located south of the Rhone river valley in Valais in south-western Switzerland (Table 1). Climatologically, all the summits investigated are located south of the inner alpine dry valleys with a continental climate (mean annual temperatures: 0.7°C and mean annual precipitation: 750 mm^1^). The summits are located at the alpine-nival ecotone spanning an elevational gradient of 850 m (between 2360 and 3212 m a.s.l.). The summits of region SN1 have calcareous (dolomite) parent material, the other seven summits have siliceous parent material (Table 1). The survey area included a substantial range of high alpine vegetation types, climatic conditions and soil properties (Supplementary Figure S1 and Supplementary Data S1).

**Table 1:** Overview of GLORIA summit locations in the Swiss Alps.

Region	Region code	Summit	Summit code	Elevation [m a.s.l.]	Latitude (N)	Longitude (E)	Parent material
Swiss National Park	SN1	Munt Buffalora	MBU	2438	46° 38′ 19″	10° 14′ 37″	Calcareous
Swiss National Park	SN1	Munt Chavagl	MCH	2542	46° 38′ 39″	10° 14′ 03″	Calcareous
Swiss National Park	SN1	Piz Murter	PMU	2836	46° 38′ 45″	10° 08′ 30″	Calcareous
Swiss National Park	SN2	Mot sper Chamana Sesvenna	MCS	2424	46° 44′ 08″	10° 25′ 43″	Siliceous
Swiss National Park	SN2	Minschuns	MIN	2519	46° 38′ 44″	10° 20′ 17″	Siliceous
Swiss National Park	SN2	Mot dal Gajer	MDG	2797	46° 41′ 40″	10° 19′ 52″	Siliceous
Valais	VAL	La Ly	LAL	2360	46° 01′ 51″	07° 14′ 57″	Siliceous
Valais	VAL	Mont Brulé	BRU	2550	46° 01′ 14″	07° 12′ 05″	Siliceous
Valais	VAL	Pointe du Parc	PAR	2989	45° 59′ 53″	07° 13′ 55″	Siliceous
Valais	VAL	Pointe de Boveire	BOV	3212	45° 59′ 40″	07° 14′ 24″	Siliceous


The floristic composition of these GLORIA summits was surveyed for the third time in 2015 (after 2001–2003 and 2008–2010) according to the GLORIA project’s standard protocol (Pauli et al., 2015). On each summit, parallel to the vegetation assessment within permanent 1 m × 1 m plots at the four corners of a 3 m × 3 m grid situated in each cardinal direction (i.e., east, south, west, north), 5 m below the highest summit point, four soil cores of 10 cm^3^ were taken per vegetation plot (at 10 cm distance from the plots) after removing the plant litter surface. Soil samples were stored on ice during the transport, kept at 4°C in the laboratory and were processed within 48 h. In all plots, all vascular plant species were recorded and the percentage of cover of each species, as well as of vascular plants, bryophytes, lichen and unvegetated surface was visually estimated following Pauli et al. (2015). In the centre of each 3 m × 3 m grid a data logger recorded the soil temperature 10 cm below the surface at hourly intervals. For all the summits, the inventories of plant communities and collection of soil samples were completed between July and August 2015.

### Basic Soil Characteristics

The fresh soil samples were sieved through a 2-mm mesh sieve and visible roots and stones were removed carefully by hand. Sieved soils were divided into two subsamples. One was stored at 4°C to determine the physical and chemical properties while the other was stored at -80°C prior to DNA extraction. Subsamples were dried at 105°C for 48 h and reweighed to obtain the gravimetric sieved soil water content. Soil texture was determined by the hydrometer technique according to Gee and Bauder (1986). The pH was measured in a 0.01 M CaCl_2_ solution with a soil-extractant ratio of 1:2 using a glass electrode linked to a pH metre (FEP20-FiveEasy Plus, Mettler- Toledo GmbH, Switzerland). Around 2 g of well-homogenised soil was milled with a Teflon ball mill. Carbon and nitrogen contents were measured in ground samples with an automated elemental analyser/continuous flow isotope ratio mass spectrometer (Euro-EA, Hekatech GmbH, Germany, interfaced with a Delta-V Advanced IRMS, Thermo GmbH, Germany). For all samples containing carbonates with pH values higher than 6.0, soil organic carbon (SOC) contents were measured after removing inorganic C using HCl vapour (Walthert et al., 2010). All the soil variables were calculated on a soil dry weight basis.

### DNA Extraction, PCR Amplification and High Throughput Sequencing

Total genomic DNA was extracted from approximately 250 mg of soil per sample using the Power Soil DNA Isolation Minikit (Qiagen, Hilden, Germany) according to the manufacturer’s protocol. DNA was quantified with PicoGreen (Invitrogen, Carlsbad, CA, United States). The V3-V4 region of the prokaryotic small-subunit (16S) rRNA and the internal transcribed spacer region 2 (ITS2) of the eukaryotic (fungal and some groups of protists and green algae) were amplified using primers and conditions previously described in Frey et al. (2016) with 20 ng of template DNA. PCRs were run in triplicates and pooled. The pooled and purified amplicons were sent to the Génome Québec Innovation Center at McGill University (Montreal, QC, Canada) for barcoding using the Fluidigm Access Array technology (Fluidigm) and paired-end sequencing on the Illumina MiSeq v3 platform (Illumina, Inc., San Diego, CA, United States). Raw sequences have been deposited in the NCBI Sequence Read Archive under the BioProject accession number PRJNA509562.

### Sequence Quality Control, OTU Clustering and Taxonomic and Functional Assignments

Quality filtering, clustering into operational taxonomic units (OTUs) and taxonomic assignment were performed as described previously by Frey et al. (2016) and Frossard et al. (2018), with the modification of using the SILVA database. In brief, a customised pipeline largely based on UPARSE (Edgar, 2013) implemented in USEARCH v. 9.2 (Edgar, 2010) was used. Sequences were dereplicated discarding singletons and clustered into OTUs with 97% identity (Edgar, 2013). Quality-filtered reads were mapped on the filtered set of centroid sequences and taxonomic classification of prokaryotic and fungal sequences was conducted querying against customised versions of SILVA (Quast et al., 2013) and UNITE (Nilsson et al., 2018). OTUs identified as mitochondria and chloroplasts were removed prior to data analysis. Fungal functional guilds were assigned within the five most abundant guilds, namely Ectomycorrhizal fungi, Lichenized fungi, Undefined saprotrophs, Wood saprotrophs, and Plant pathogens, using an open annotation tool (FUNGuild) according to Nguyen et al. (2016). Only the guild assignment with “highly probable” confidence rankings was accepted.

### Data Analysis

For analysis of microbial α-diversity, observed richness (number of OTUs) and Shannon diversity index were estimated based on OTU abundance matrices rarefied to the lowest number of sequences. The relationships between abiotic and biotic variables, geographical parameters (elevation, aspect) and α-diversity indices were tested using linear mixed-effects models in order to control for nested effects and non-independent data points. For this the function *lme* in the R package *nlme* (v3.1.137; Pinheiro et al., 2018) was used with region, summit and aspect as random effects, and the maximum likelihood method to assess the significance of the fixed effects. In all models, data was normalised, where necessary, to account for differing magnitudes. Likewise, the same method was used for testing the relationships of relative abundances of individual phyla and classes against elevation and soil pH. For all linear models, assumptions for homoscedasticity and normality of residuals were tested and, where necessary, transformation (logarithmic or Tukey’s ladder of powers) of response variables was performed. Variables with known influences on plant and soil microbial communities were selected manually to build multivariate models of α-diversity indices. The AIC (Akaike Information Criteria) was used to evaluate model performance and included interaction terms of individual variables with both elevation and aspect. Covariance among variables was first tested to exclude highly collinear predictors (Supplementary Figure S2).

Bray–Curtis dissimilarities were calculated based on square-root transformed relative abundances of OTUs. Differences in community structure (β-diversity) between different summits and aspects were assessed by conducting a permutational ANOVA (PERMANOVA, number of permutations = 9,999) with the function *adonis* implemented in the *vegan* package (v2.5.3; Oksanen et al., 2018). We used PERMANOVA to assess the influence of the abiotic and biotic variables on bacterial and fungal community structure with strata to constrain permutations to within summits (random term). Principal coordinates analysis (PCoA) ordinations of microbial community structure were calculated using the *ordinate* function implemented in the R package *phyloseq* (v1.26.0; McMurdie and Holmes, 2013). Indicator species analysis was performed using the *multipatt* function implemented in the *indicspecies* package (v1.7.6; De Cáceres and Legendre, 2009) with 9,999 permutations and allowing combinations between habitats. The correlation index (*r.g*) was used to identify characteristic OTUs associated with a particular type of parent material.

To examine the relationship between plant and microbial β-diversity, we compared Bray–Curtis dissimilarity matrices for plants with those for bacteria and fungi using Mantel tests and Spearman correlations in the *vegan* package. To avoid pseudo-replication, we averaged the dissimilarities by summit. Final pair-wise comparisons between summits were averaged from sample-level dissimilarities. In order to calculate the plant β-diversity for the aforementioned analysis we used the vascular plant richness data and the corresponding plant taxonomy. All statistical analyses were performed using R (v.3.5.1; R Core Team, 2018) and all graphs were generated with the *ggplot2* package (v3.1.0; Wickham, 2016), unless specified otherwise.

## Results

### Characterisation of Microhabitats on Summits

Mean soil temperatures at a depth of 5 cm in both winter (December–February) and summer (June–August) decreased significantly with elevation (Supplementary Figure S3, Supplementary Table S1, and Supplementary Data S1). Across all summits, mean soil temperatures in winter (MWST) ranged from -9.7°C (northern aspect) to 1.1°C (southern aspect), whereas mean soil temperatures in summer (MSST) ranged from 3.9°C (northern aspect) to 12.9°C (southern aspect). The most extreme soil temperatures recorded were -16.7°C at MDG (2797 m a.s.l., northern aspect) and 27.4°C at the highest summit (BOV, 3212 m a.s.l., southern aspect). Within a single summit, the difference between the mean temperatures of the coldest soils on northern aspects and the warmest soils on southern aspects was as high as 4.5°C in the summer months (Supplementary Data S1).

The soil pH ranged from 3.8 (on siliceous parent material) to 7.6 (on calcareous parent material; Supplementary Data S1). SOC content ranged from 0.3 to 30.5% (mean 6.9%); higher summits contained less soil carbon than lower summits. The C:N ratio across all summits varied between 9.1 and 19.7 (mean 13.2). Soil texture varied considerably among summits with average sand contents of 50 ± 21% (ranging from 20 to 88%), silt contents of 42 ± 22% (ranging from 6 to 72%) and clay contents of 8 ± 6% (ranging from 2 to 31%; Supplementary Figure S1).

Vegetation characteristics on summits differed among elevations and aspects (Supplementary Data S1). The richness of vascular plant species decreased significantly with increasing elevation (Supplementary Table S1), and eastern and southern aspects were richer in plant species (20 ± 11 and 16 ± 10, respectively) than the northern and western aspects (12 ± 9 and 11 ± 7, respectively). This variation in plant species richness corresponded to warmer mean temperatures during the growing seasons on the southern and eastern aspects compared to the northern and western aspects. Plant species richness was generally higher on siliceous than on calcareous parent material. Vascular plant cover was more abundant than bryophytes and lichens on the lower summits but less abundant on the highest summits (Supplementary Figure S1 and Supplementary Data S1). DNA content, as a proxy for total biomass, significantly decreased with increasing elevation (Supplementary Figure S3 and Table S1). We observed a similar trend for SOC with elevation (Supplementary Figure S3 and Table S1) and furthermore a significant positive correlation between DNA content and SOC (Supplementary Figure S3).

### Taxonomic Composition of Prokaryotic and Fungal Communities

After quality filtering and singleton removal, a total of 2,090,694 prokaryotic and 2,612,421 fungal sequences remained for community analysis. Over the 135 samples, this corresponds to an average of 15,487 ± 5,454 prokaryotic and 19,351 ± 3,732 fungal sequences per sample. Sequence clustering yielded 10,465 prokaryotic OTUs (1,718 ± 727 per sample) and 6,291 fungal OTUs (399 ± 96 per sample) from the 10 summits and four aspects, respectively. A total of 59 archaeal OTUs accounting for 0.70% (14,561 sequences) of the prokaryotic sequences were identified. *Thaumarchaeota* (14 OTUs, 10,552 sequences), *Euryarchaeota* (15 OTUs, 2,280 sequences), *Woesearchaeota* (18 OTUs, 320 sequences) and *Parvarchaeota* (2 OTUs, 16 sequences) were identified at the phylum level. The remaining 10 OTUs were not classified at phylum level. Since archaeal sequences were a minor part of the total prokaryotic sequences they were not analysed further.

**FIGURE 1:**
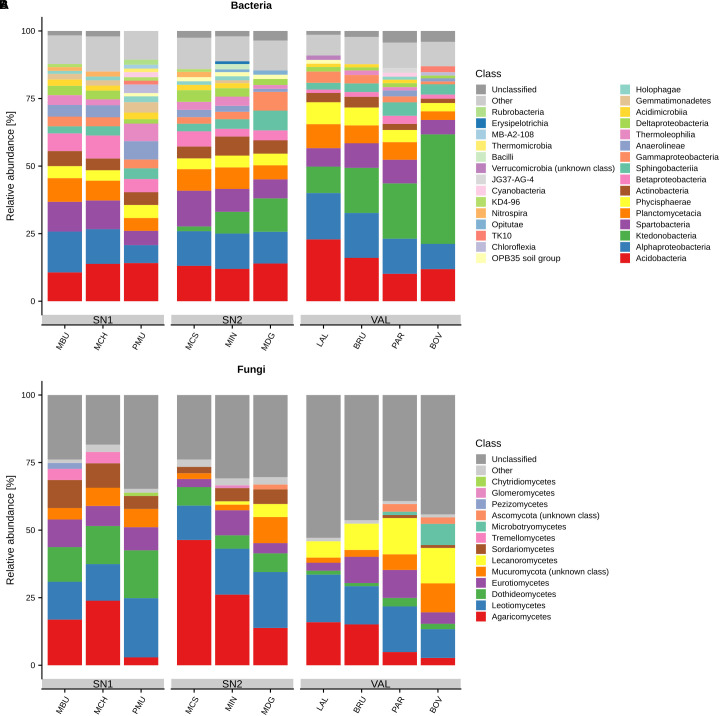
Relative abundances of **(A)** bacterial and **(B)** fungal most abundant classes (>1%) across different summits. “Other” represents all classes with relative abundances < 1%. Summits are grouped by regions and ordered by increasing elevation (left–right) within. Region abbreviations: SN1 = Swiss National Park, calcareous parent material; SN2 = Swiss National Park, siliceous parent material; VAL = Valais, siliceous parent material. Summit abbreviations: MBU = Munt Buffalora; MCH = Munt Chavagl; PMU = Piz Murter; MCS = Mot sper Chamana Sesvenna; MIN = Minschuns; MDG = Mot dal Gajer; LAL = La Ly; BRU = Mont Brulé; PAR = Pointe du Parc; BOV = Pointe de Boveire.

We identified 10,406 bacterial OTUs which could be assigned to 40 unique phyla, 92 classes and 379 genera (Supplementary Figure S4). *Proteobacteria* (24.3% relative abundance, 1,993 OTUs), *Acidobacteria* (14.9%, 737 OTUs), *Chloroflexi* (13.7%, 1,106 OTUs), *Planctomycetes* (12.4%, 1729 OTUs), *Verrucomicrobia* (12%, 611 OTUs), *Actinobacteria* (9.4%, 592 OTUs), *Bacteroidetes* (4.5%, 631 OTUs), *Parcubacteria* (2.2%, 1097 OTUs) and *Gemmatimonadetes* (1.3%, 187 OTUs) were the most abundant bacterial phyla (Supplementary Figures S4, S5). The most abundant classes (relative abundance > 1%), and their variation across summits, are depicted in Figure 1. Among the *Proteobacteria*, class *Alpha* accounted for 13.3% (590 OTUs), *Beta* for 4.5% (269 OTUs), *Gamma* for 3.7% (359 OTUs) and *Delta* for 2.8% (733 OTUs) of the total abundance. The success of taxonomic assignment decreased at lower taxonomic levels, revealing 8,111 OTUs (78%), 7,153 OTUs (68%), 5,235 OTUs (50%), 2,499 OTUs (24%) and 166 OTUs (1.6%) that were identified at the class, order, family, genus and species levels, respectively (Supplementary Figure S4). The most abundant OTU was *Bradyrhizobium* within the *Proteobacteria* (2.7%; 56,313 total sequences). The 10 most abundant classified bacterial genera were *Bradyrhizobium* (2.7%), *Candidatus Xiphinematobacter* (2.5%), *Ktedonobacter* (2.3%), *Bryobacter* (2%), *Chthoniobacter* (1.8%), *Candidatus Solibacter* (1.7%), *Acidothermus* (1.5%), *Pir4 lineage* (0.95%), *Mycobacterium* (0.9%) and *Blastocatella* (0.8%). A complete list of all bacterial OTUs including taxonomic assignment, the number of sequences and abundance information can be found in Supplementary Data S2.

The 6,291 obtained fungal OTUs could be assigned to six phyla, 26 classes and 421 genera (Supplementary Figure S4). Around 31% of the total sequences could not be classified at the phylum level. *Ascomycota* (56.7%, 2,940 OTUs) and *Basidiomycota* (23.5%, 1,208 OTUs) were the predominant phyla followed by *Mucoromycota*, *Glomeromycota* and *Chytridiomycota* all accounting for 4.9% (195 OTUs; Supplementary Figures S4, S5). The class that presented the highest relative abundance and OTU richness was that of *Agaricomycetes* (889 OTUs, 547,808 sequences, 89.3% of total *Basidiomycota*; Figure 1). It was followed by three *Ascomycota* classes: *Leotiomycetes* (510 OTUs), *Dothideomycetes* (367 OTUs) and *Eurotiomycetes* (329 OTUs). Approximately 76% of the total OTUs could not be classified at the genus level. The 10 most abundant classified fungal genera were *Mortierella* (4%), *Hygrocybe* (3.2%), *Inocybe* (2.9%), *Rhizoscyphus* (2.4%), *Capronia* (1.7%), *Geomyces* (1.5%), *Parmelia* (1.42%), *Tomentella* (1.3%), *Naganishia* (1.25%; formerly lumped into the genus *Cryptococcus*; Liu X.Z. et al., 2015; Schmidt et al., 2017), and *Leohumicola* (1.2%). All detected fungal OTUs, taxonomic assignment, the number of sequences and abundance information are shown in Supplementary Data S3.

### Bacterial and Fungal α-Diversity

**FIGURE 2:**
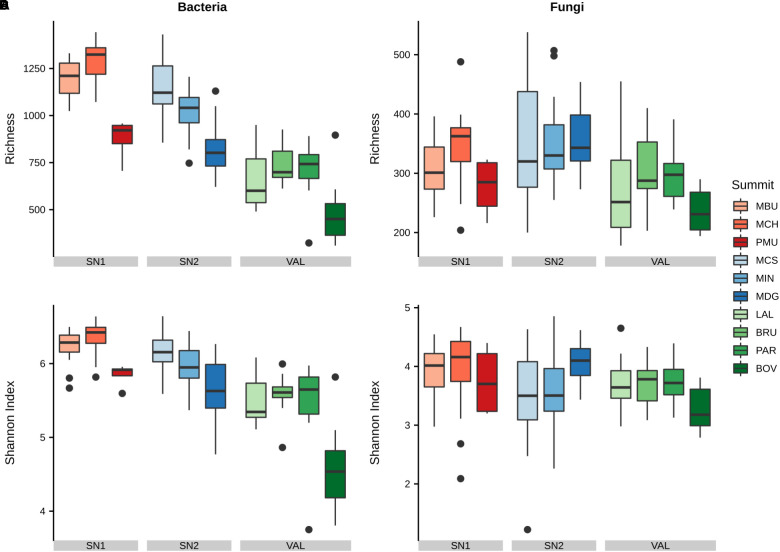
Variation of α-diversity of bacterial and fungal communities at different summits. Shown are **(A)** bacterial richness, **(B)** fungal richness, **(C)** bacterial Shannon index and **(D)** fungal Shannon index. Summits are grouped by regions and ordered by increasing elevation (left–right) within. Region abbreviations: SN1 = Swiss National Park, calcareous parent material; SN2 = Swiss National Park, siliceous parent material; VAL = Valais, siliceous parent material. Summit abbreviations: MBU = Munt Buffalora; MCH = Munt Chavagl; PMU = Piz Murter; MCS = Mot sper Chamana Sesvenna; MIN = Minschuns; MDG = Mot dal Gajer; LAL = La Ly; BRU = Mont Brulé; PAR = Pointe du Parc; BOV = Pointe de Boveire.

Generally, bacterial α-diversity was higher in soils on calcareous parent material in region SN1 and lower in soils on siliceous parent material in region VAL. Within each region, bacterial richness was lowest at the highest elevation (SN1: PMU; SN2: MDG; VAL: BOV; Figure 2 and Supplementary Data S1). The bacterial Shannon diversity followed similar trends as richness. Furthermore, in most cases the western aspect showed the highest bacterial α-diversity (Supplementary Figure S6).

Fungal α-diversity was in general lower in the VAL region, however, these trends were less pronounced than for bacterial α-diversity. In the VAL region (siliceous parent material) western aspects showed the highest fungal richness and the northern aspects the lowest richness, whereas in region SN1 (calcareous parent material) we observed the opposite trend (Supplementary Figure S6 and Supplementary Data S1). At large, fungal richness was lower than bacterial richness at all summits. Among the fungal guilds we found that undefined saprotrophs (7.2%), lichenized fungi (4.7%), ectomycorrhizal fungi (4.0%), wood saprotrophs (1.3%) and plant pathogens (0.5%) were the dominant functional guilds with a “highly probable” classification (Nguyen et al., 2016). However, no clear patterns in Shannon diversity of the different guilds with elevation were found apart from wood saprotrophs, which exhibited a significant decrease in SN2 (Supplementary Figure S7).

### Linking α-Diversity to Topographical andEnvironmental Variables

**FIGURE 3:**
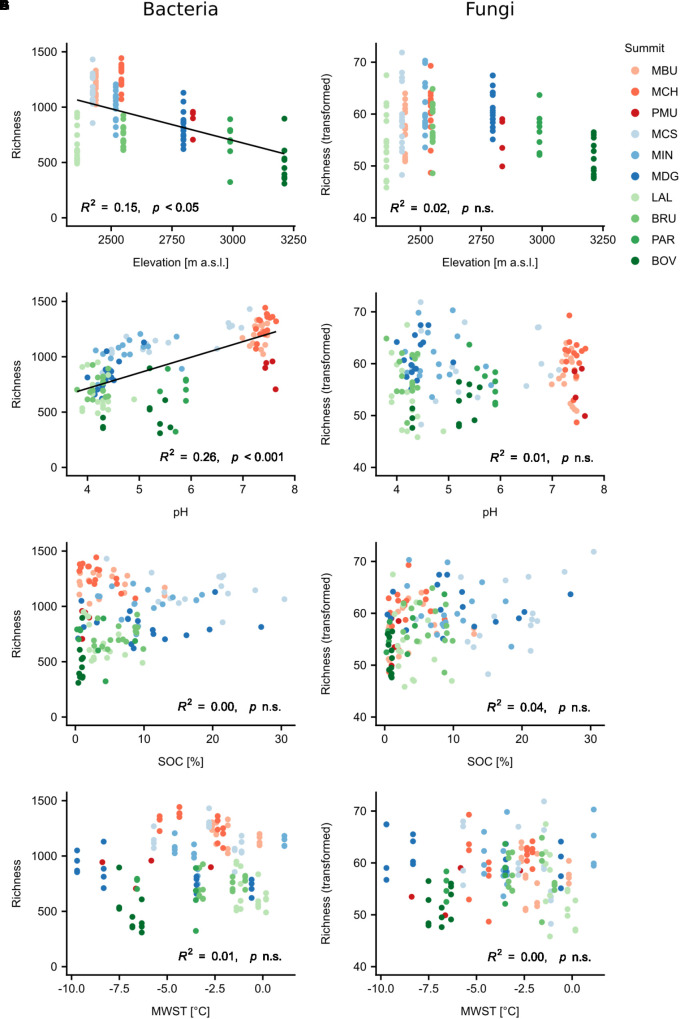
Relationships of bacterial and fungal observed richness with elevation and selected environmental variables **(A–H)**. Regression lines were fitted using linear mixed-effects models with region/summit/aspect as random effects. Fungal observed richness was transformed using Tukey’s ladder of powers. Abbreviations: SOC, soil organic carbon; MWST, mean winter soil temperature; n.s., not significant. Summit abbreviations: MBU = Munt Buffalora; MCH = Munt Chavagl; PMU = Piz Murter; MCS = Mot sper Chamana Sesvenna; MIN = Minschuns; MDG = Mot dal Gajer; LAL = La Ly; BRU = Mont Brulé; PAR = Pointe du Parc; BOV = Pointe de Boveire. Colours correspond to regions: Red = SN1, Swiss National Park, calcareous parent material; Blue = SN2, Swiss National Park, siliceous parent material; Green = VAL, Valais, siliceous parent material.

To determine the influence of elevation, aspect and potential environmental drivers and their interactions on the diversity patterns of bacteria and fungi, we applied linear mixed-effects regression analysis (Figure 3, Table 2, and Supplementary Tables S2, S3). For all models, the nested groupings of region, summit and aspect were selected as random effects to account for non-independent data. Bacterial richness and Shannon diversity were found to decrease with increasing elevation (richness: normalised slope = -0.34, *F* = 7.23, *p* < 0.05; similar trend for Shannon), however fungal richness and Shannon diversity showed no significant change (Figure 3). When accounting for nested effects, aspect alone revealed no significant effect on the α-diversity of both taxa. However, we found the interaction of elevation and aspect to play a slight, but significant role for bacterial richness (*F* = 4.87, *p* < 0.01). Bacterial richness decreased with higher elevation on all aspects except for the northern one (north: *p* > 0.05), with the western aspect having the strongest negative impact (norm. slope: -0.71, p_lme_ < 0.001). Effects of both mean summer and winter soil temperatures on α-diversity were observed for fungi and not bacteria, however only in interaction with elevation (MSST × elevation: norm. slope = 0.19, *F* = 5.23, *p* < 0.05; MWST × elevation: norm. slope = 0.31, *F* = 9.38, *p* < 0.01; the trend for Shannon was slightly weaker). The influences of SOC, total nitrogen (TN) and C:N ratio varied between fungi and bacteria, and mostly exhibited significant effects also only in interaction with elevation (Supplementary Tables S2, S3). As the strongest individual predictor of bacterial α-diversity we identified soil pH (richness: norm. slope = 0.48, *F* = 16.7, *p* < 0.001; similar finding for Shannon), which conversely had no significant effect on fungal α-diversity. The change of vegetation composition (bryophytes, lichen, vascular plants), as well as plant richness, in interaction with elevation influenced both bacterial and fungal α-diversities with the effect being more evident for fungi. These effects, in particular of plant richness, were found to be stronger for the Shannon diversity of both taxa (bacteria: norm. slope = 0.29, *F* = 8.79, *p* < 0.01; fungi: norm. slope = 0.33, *F* = 14.46, *p* < 0.001). In general, soil texture mainly influenced fungal richness and Shannon diversity with silt being the strongest negative predictor for fungal richness (norm. slope = -0.34, *F* = 7.29, *p* < 0.01).

**Table 2:** Effects of elevation, aspect and environmental variables on bacterial and fungal α-diversities analysed with multivariate linear mixed-effects regression.

	Fixed effects	DF_num_	DF_den_	*F*	*p*
**Bacteria**					
*S_obs_*	Intercept	1	97	0.00	0.9570
	pH	1	97	33.83	<0.0001
	Aspect	3	16	3.13	0.0547
	Elevation × Aspect	4	16	8.34	0.0008
	MWST × Aspect	4	16	5.64	0.0050
*R^2^* marginal: 64.18%, *R^2^* conditional: 82.23%					
*Shannon*					
	Intercept	1	96	1218.05	<0.0001
	pH	1	96	96.15	<0.0001
	SOC	1	96	23.50	<0.0001
	Aspect	3	16	9.67	0.0007
	Elevation × Aspect	4	16	12.98	0.0001
	MWST × Aspect	4	16	11.38	0.0001
*R^2^* marginal: 74.81%, *R^2^* conditional: 76.18%					
**Fungi**					
*S_obs_*	Intercept	1	93	1624.34	<0.0001
	TN	1	93	18.86	<0.0001
	Vegetation cover × Elevation	1	93	23.55	<0.0001
	Vegetation cover × Aspect	3	93	6.41	0.0005
	MWST × Aspect	4	23	4.71	0.0063
*R^2^* marginal: 39.47%, *R^2^* conditional: 39.60%					
*Shannon*					
	Intercept	1	94	1,494.35	<0.0001
	Aspect	3	24	2.51	0.0830
	Vegetation cover × Elevation	1	94	18.78	<0.0001
	Vegetation cover × Aspect	3	94	5.52	0.0016
*R^2^* marginal: 25.37%, *R^2^* conditional: 25.37%					


*Shown is *F*- statistic of ANOVA results for linear mixed-effects models (random effects: region/summit/aspect). *R^*2*^* indicates the proportion of variation explained by the environmental parameters; marginal *R^*2*^* is the variation explained by all fixed effects together; conditional *R^*2*^* is the variation explained by both fixed and random effects (see section “Materials and Methods”). Models were selected using the AIC criterion. Fungal observed richness as well as bacterial and fungal Shannon diversity indices were transformed using Tukey’s ladder of powers. S_*obs*_, observed richness; Shannon, Shannon diversity; DF, degrees of freedom; num, numerator; den, denominator; MWST, mean winter soil temperature; SOC, soil organic carbon; TN, total nitrogen*.

The best combination of environmental predictors explaining bacterial richness (in order of decreasing relative importance) were soil pH, the interaction between elevation and aspect, the interaction between MWST and aspect, and aspect alone (marginal *R*^2^= 64.18%; Table 2). The best model explaining bacterial Shannon diversity included SOC content as the second strongest predictor after soil pH, in addition to the aforementioned predictors for bacterial richness (marginal *R*^2^ = 74.81%; Table 2). In contrast, the best model for fungal richness (marginal *R*^2^= 39.47%) included the interaction of vegetation cover with elevation, followed by TN and the interactions of both vegetation cover and MWST with aspect. Fungal Shannon diversity was best explained by the interactions of vegetation cover with elevation and aspect, respectively, and by aspect itself (marginal *R*^2^= 25.37%; Table 2). In general, these linear mixed-effects models were able to explain considerably more of the variation of bacterial richness and Shannon diversity than of fungal diversity patterns.

### Bacterial and Fungal β-Diversity

**FIGURE 4:**
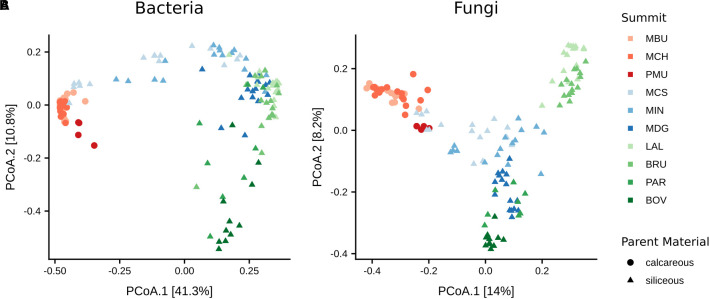
Principal coordinate analysis (PCoA) of **(A)** bacteria and **(B)** fungal β-diversities based on Bray–Curtis distance matrices. Distances between symbols on the ordination plot reflect relative dissimilarities in community structures. The variation in microbial community structures explained by each PCoA axis is given in parentheses.

To examine the variation of the bacterial community structures with respect to summit, aspect and their interactions we performed permutational multivariate analysis of variance (PERMANOVA) based on Bray–Curtis dissimilarity. The analysis revealed the effect of summit being more influential than that of aspect (summit: *F*_(9,98)_= 23.3, *p* < 0.001, *R*^2^ = 56.6%; aspect: *F*_(3,98)_ = 2.7, *p* < 0.001, *R*^2^ = 2.2%). The interaction of both summit and aspect also only had a marginal effect (*F*_(24,98)_ = 2.3, *p* < 0.001, *R*^2^ = 14.8%). To visualise the community structure we used PCoA based on the Bray–Curtis metric (Figure 4). In total, 52.1% of the variation in the bacterial community structure could be explained by the two axes. We found that the type of parent material predominantly structured the bacterial communities. Bacterial communities on calcareous parent material clustered at a noticeable distance from bacterial communities grown on siliceous parent material. In addition, bacterial communities on siliceous parent material were more widely dispersed, leading to distinct but partially overlapping clusters of the geographic regions SN2 and VAL.

As with bacteria, the influence of summit was stronger than aspect on fungal β-diversity (summit: *F*_(9,98)_= 11.4, *p* < 0.001, *R*^2^ = 38.6%; aspect: *F*_(3,98)_ = 3.0, *p* < 0.001, *R*^2^ = 3.4%). Likewise, the impact of the interaction of elevation and aspect was less (*F*_(24,98)_ = 2.3, *p* < 0.001, *R*^2^ = 20.9%) than that of summit alone. 22.2% of the variation of the fungal community structure could be explained by the two axes of the PCoA ordination (Figure 4). Similar to bacterial communities, fungal communities in soils on calcareous parent material in SN1 were separated along the first axis from those on siliceous parent material. Fungal communities in the VAL region were clearly separated along the second axis with summits at higher elevations (PAR, 2989 m a.s.l.; BOV, 3212 m a.s.l.) grouping separate from those at lower elevations (LAL, 2360 m a.s.l.; BRU, 2550 m a.s.l.).

### Linking β-Diversity to Environmental Variables

To identify individual environmental parameters explaining the bacterial and fungal community structure we performed PERMANOVA based on Bray–Curtis dissimilarity (Table 3). We found that the highest proportion of the variation in the bacterial community structure could be attributed to pH (36.1%), followed by vegetation cover (10.2%) and elevation (9.3%). SOC and TN explained only a small proportion (each < 5%) of the bacterial community structure, despite being statistically significant. Mean soil temperatures in summer and winter, as well as soil texture and C:N ratio did not significantly contribute to the structuring of the bacterial communities. Looking at fungal community structure, the predictive power of the individual models were overall less, however similar parameters were found. Soil pH (12.2%), elevation (7.2%) and vegetation cover (6.6%) accounted for the highest contributions to community structure, with several other variables, notably MSST, MWST, SOC and TN having significant, however, smaller contributions.

**Table 3:** Effects of elevation and selected individual environmental variables on bacterial and fungal community structures assessed by permutational multivariate analysis of variance (PERMANOVA).

Explanatory variable	Bacteria		Fungi	
	*F*_(1,134)_	Variation	*F*_(1,134)_	Variation
Elevation	**13**.57***	9.3%	**10**.22***	7.2%
Soil pH	**75**.20***	36.1%	**18**.43***	12.2%
MWST	4.51 n.s.	3.3%	**7**.24***	5.2%
MSST	8.71 n.s.	6.2%	**8**.74***	6.2%
SOC	**6**.42***	4.6%	**5**.76***	4.2%
TN	**6**.10***	4.4%	**5**.75***	4.1%
C:N	7.00 n.s.	5.0%	**3**.38**	2.5%
Vegetation cover	**15**.08***	10.2%	**9**.45***	6.6%
Sand	6.56 n.s.	4.7%	7.95 n.s.	5.6%
Silt	9.26 n.s.	6.5%	9.02 n.s.	6.3%
Clay	9.12 n.s.	6.4%	**4**.23*	3.1%


*F-ratio tests were conducted to assess the effects of environmental variables on bacterial and fungal community structures. For all variables (except for elevation) summit was used as a blocking variable (strata = summit) to restrict permutations to within summits. The indices are the degrees of freedom and residuals for each factor. Significant variables are in bold. Significance levels: n.s. (not significant), ^∗^*p* < 0.05, ^∗∗^*p* < 0.01, ^∗∗∗^*p* < 0.001. Permutations: 9,999. MWST, mean winter soil temperature; MSST, mean summer soil temperature; SOC, soil organic carbon; TN, total nitrogen; C:N, carbon to nitrogen ratio*.

### Influence of Elevation and pH on Relative Abundances of Microbial Phyla and Classes

As elevation and soil pH were among the strongest parameters influencing microbial community structure, we examined their influence also on the relative abundances (>1%) of major bacterial and fungal phyla (Table 4). The strongest negative relationships with elevation were exhibited by bacteria of the phyla *Planctomycetes* (norm. slope = -0.51, *F* = 71.4, *p* < 0.001) and *Proteobacteria* (norm. slope = -0.46, *F* = 18.3, *p* < 0.01), whose relative abundances decreased significantly with increasing elevation. A negative relationship with elevation was also observed for abundances of *Verrucomicrobia* (norm. slope = -0.28, *F* = 15.1, *p* < 0.01), albeit not as strong as for the aforementioned phyla. In contrast, the relative abundance of *Chloroflexi* (norm. slope = 0.49, *F* = 46.8, *p* < 0.001), *Gemmatimonadetes* (norm. slope = 0.19, *F* = 7.9, *p* < 0.05) and *Bacteroidetes* (norm. slope = 0.16, *F* = 7.7, *p* < 0.05) showed significant increases with elevation. Changes in soil pH in particular affected the relative abundance of *Gemmatiomonadetes* (norm. slope = 0.33, *F* = 22.5, *p* < 0.001), *Parcubacteria* (norm. slope = 0.37, *F* = 13.9, *p* < 0.001) and *Proteobacteria* (norm. slope = 0.33, *F* = 11.4, *p* < 0.01), which all increased significantly with increasing pH (Table 4), and *Chloroflexi*, which decreased in abundance slightly, albeit significantly, with increasing soil pH (norm. slope = -0.22, *F* = 5.9, *p* < 0.05).

**Table 4:** Changes of the relative abundances of the most abundant bacterial and fungal phyla (>1%) with elevation and soil pH analysed with linear mixed-effects regression.

	Elevation			Soil pH		
Phylum	Norm. slope	Direction of change	*F*	Norm. slope	Direction of change	*F*
**Bacteria**						
*Acidobacteria*	-0.18	↓	3.2 n.s.	-0.10	–	2.0 n.s.
*Actinobacteria*	-0.20	↓	4.3 n.s.	0.04	–	0.2 n.s.
*Bacteroidetes*	0.16	↑	7.7*	0.05	–	0.9 n.s.
*Chloroflexi*	0.49	↑	46.8***	-0.22	↓	5.9*
*Gemmatimonadetes*	0.19	↑	7.9*	0.33	↑	22.5***
*Parcubacteria*	-0.15	↓	2.4 n.s.	0.37	↑	13.9***
*Planctomycetes*	-0.51	↓	71.4***	0.08	–	0.9 n.s.
*Proteobacteria*	-0.46	↓	18.3**	0.33	↑	11.4**
*Verrucomicrobia*	-0.28	↓	15.1**	-0.07	–	0.8 n.s.
**Fungi**						
*Ascomycota*	-0.12	–	0.9 n.s.	-0.08	–	0.5 n.s.
*Basidiomycota*	-0.31	↓	8.2*	-0.01	–	0.0 n.s.
*Mucoromycota*	0.36	↑	16.1**	0.05	–	0.3 n.s.


*Shown is *F*-statistic of ANOVA results for linear mixed-effects models (random effects: region/summit/aspect). Direction of change: increase (↑), decrease (↓), or no clear pattern (–) in slope of regression line. Slope of regression line is normalised to account for differing units. Significance levels: n.s. (not significant), ^∗^*p* < 0.05, ^∗∗^*p* < 0.01, ^∗∗∗^*p* < 0.001. Relative abundances of bacterial and fungal phyla were transformed using log transformation or Tukey’s ladder of powers if model assumptions were not fulfilled*.

The two bacterial classes that exhibited the strongest responses, both negative, to increasing elevation were *Planctomycetacia* (norm. slope = -0.49, *F* = 49.5, *p* < 0.001) and *Alphaproteobacteria* (norm. slope = -0.48, *F* = 20.0, *p* < 0.01; Table 5). In contrast, the relative abundance of *Ktedonobacteria*, of the phylum *Chloroflexi*, increased at higher elevations (norm. slope = 0.27, *F* = 12.6, *p* < 0.05). Soil pH in turn had a strong negative effect on *Ktedonobacteria* (norm. slope = -0.53, *F* = 44.6, *p* < 0.001), whereas *Betaproteobacteria* (norm. slope = 0.42, *F* = 36.8, *p* < 0.001) significantly increased with higher pH. None of the other major bacterial classes showed a significant relationship with soil pH (Table 5).

**Table 5:** Changes of the relative abundances of the most abundant bacterial and fungal classes (>1%) with elevation and soil pH analysed with linear mixed-effects regression.

	Elevation			Soil pH		
Phylum	Norm. slope	Direction of change	*F*	Norm. slope	Direction of change	*F*
**Bacteria**						
*Acidobacteria*	-0.17	↓	2.4 n.s.	-0.11	↓	2.3 n.s.
*Actinobacteria*	-0.26	↓	9.6*	-0.09	–	0.8 n.s.
*Alphaproteobacteria*	-0.48	↓	20.0**	0.00	–	0.0 n.s.
*Betaproteobacteria*	0.02	–	0.0 n.s.	0.42	↑	36.8***
*Gammaproteobacteria*	-0.18	↓	3.4 n.s.	0.07	–	1.1 n.s.
*Ktedonobacteria*	0.27	↑	12.6*	-0.53	↓	44.6***
*Phycisphaerae*	-0.32	↓	12.6*	-0.03	–	0.1 n.s.
*Planctomycetacia*	-0.49	↓	49.5***	0.08	–	0.9 n.s.
*Spartobacteria*	-0.23	↓	6.0 n.s	-0.07	–	0.7 n.s.
*Sphingobacteriia*	0.2	↑	7.9*	-0.01	–	0.0 n.s.
**Fungi**						
*Agaricomycetes*	-0.42	↓	34.8**	-0.03	–	0.1 n.s.
*Dothideomycetes*	0.10	↑	3.1 n.s.	0.27	↑	11.9***
*Eurotiomycetes*	0.03	–	0.1 n.s	0.01	–	0.0 n.s.
*Lecanoromycetes*	0.17	↑	8.1*	-0.20	↓	4.5*
*Leotiomycetes*	-0.02	–	0.1 n.s.	-0.02	–	0.1 n.s.
*Sordariomycetes*	-0.02	–	0.1 n.s.	0.15	↑	2.0 n.s.
*Tremellomycetes*	-0.06	–	0.4 n.s.	0.10	–	1.1 n.s.
*Mucoromycota (unknown class)*	0.36	↑	16.1**	0.05	–	0.3 n.s.


*Shown is *F*-statistic of ANOVA results for linear mixed-effects models (random effects: region/summit/aspect). Direction of change: increase (↑), decrease (↓), or no clear pattern (–) in slope of regression line. Slope of regression line is normalised to account for differing units. Significance levels: n.s. (not significant), ^∗^*p* < 0.05, ^∗∗^*p* < 0.01, ^∗∗∗^*p* < 0.001. Relative abundances of bacterial and fungal classes were transformed using log transformation or Tukey’s ladder of powers if model assumptions were not fulfilled*.

In contrast to bacteria, the effect of elevation on relative abundances of fungal phyla was overall weaker (Table 4). The abundance of *Basidiomycota* (norm. slope = -0.31, *F* = 8.2, *p* < 0.05) declined at higher elevations, whereas *Mucoromycota* (norm. slope = 0.36, *F* = 16.1, *p* < 0.01) increased. The relative abundance of *Ascomycota* exhibited no significant change with increasing elevation. None of the fungal phyla showed any significant change in relative abundances to changes in pH (Table 4).

For fungal classes, we found that the relative abundances of *Agraricomycetes* decreased strongly with increasing elevation (norm. slope = -0.42, *F* = 34.8, *p* < 0.01). We also observed a significant increase in abundance of a yet unknown class of *Mucoromycota* (norm. slope = 0.36, *F* = 16.1, *p* < 0.05) as well as *Lecanoromycetes* (norm. slope = 0.17, *F* = 8.1, *p* < 0.05). A significant increase in abundance in response to higher soil pH was observed for *Dothideomycetes* (norm. slope = 0.27, *F* = 11.9, *p* < 0.001). Conversely, *Lecanoromycetes* (norm. slope = -0.20, *F* = 4.5, *p* < 0.05) decreased in abundance at higher pH levels, albeit to a far lesser degree (Table 5).

### Relationship Between Plant and Microbial Diversities

**FIGURE 5:**
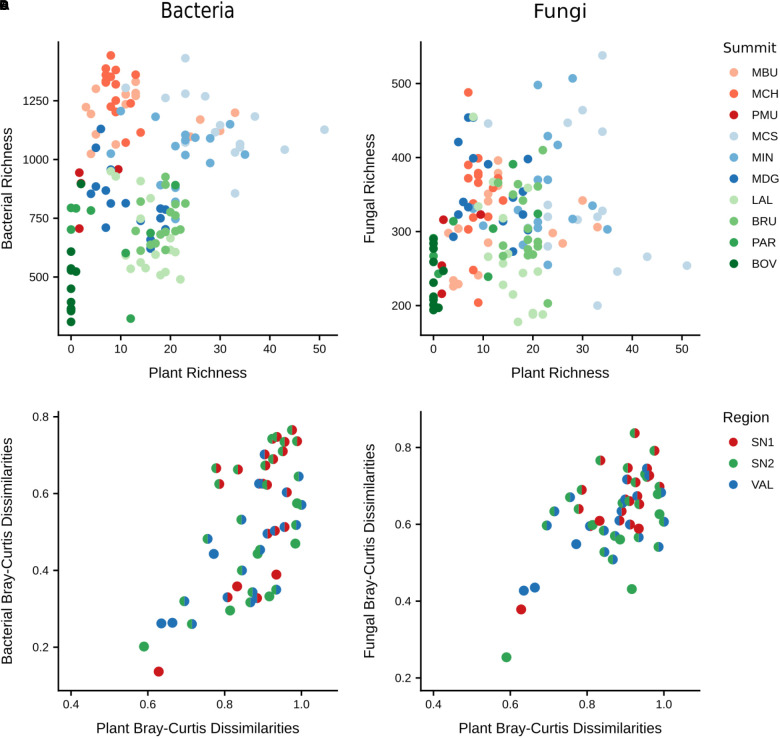
Comparison of α and β-diversities between microbial and plant communities. Upper scatterplots show relationships of plant richness with **(A)** bacterial richness (*F* = 1.04, *p* = 0.31) and **(B)** fungal richness (*F* = 0.48, *p* = 0.49). Lower plots show relationships of plant β-diversity with **(C)** bacterial β-diversity (ρ = 0.57, *p* = 0.001) and **(D)** fungal β-diversity (ρ = 0.47, *p* = 0.004). In **(C,D)** each point represents the dissimilarities (Bray–Curtis) of taxonomic composition between a pair of summits (colours represent regions of each summit). The significances of correlations of α-diversity were assessed applying ANOVA to the output of linear mixed-effects models (random effects: region/summit/aspect). The significances of the correlations for β-diversity were assessed by Mantel tests with 9,999 permutations based on Spearman’s rank method. Region abbreviations: SN1 = Swiss National Park, calcareous parent material; SN2 = Swiss National Park, siliceous parent material; VAL = Valais, siliceous parent material. Summit abbreviations: MBU = Munt Buffalora; MCH = Munt Chavagl; PMU = Piz Murter; MCS = Mot sper Chamana Sesvenna; MIN = Minschuns; MDG = Mot dal Gajer; LAL = La Ly; BRU = Mont Brulé; PAR = Pointe du Parc; BOV = Pointe de Boveire.

To examine the relationship between the soil microbiome and vascular plants, we compared microbial α- and β-diversities with those of plants (Figure 5). Both bacterial and fungal richness showed no significant relationships with plant richness (Supplementary Tables S2, S3). However, the interaction of plant richness and elevation did affect bacterial and fungal richness significantly (bacteria: *F* = 7.05, *p* < 0.01; fungi: *F* = 8.34, *p* < 0.01), indicating that plant richness positively influences microbial richness only as elevation increases. Furthermore, bacterial and fungal community structures exhibited significant positive relationships with plant community structure (Bray–Curtis dissimilarities), showing that summits with a more distinct composition of plant communities also revealed more distinct bacterial and fungal communities (bacteria ρ = 0.57, *p* = 0.001; fungi ρ = 0.47, *p* = 0.004; Figure 5).

### Bacterial and Fungal Indicator Taxa

**FIGURE 6:**
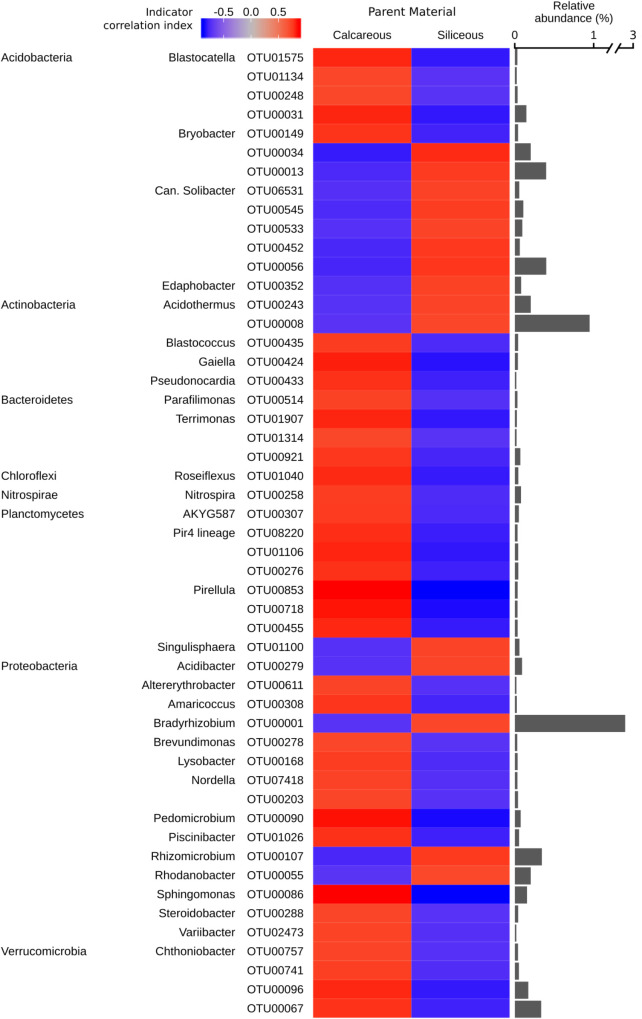
Bacterial genera identified as significant indicators (*q* < 0.05) of calcareous or siliceous parent material (strength of correlation index > 0.75), grouped by phyla. The colour of the heatmap represents the strength of indication. The bars represent the relative abundance of each indicator OTU in all the samples.*(Indicator OTUs with a relative abundance of < 0.01% are not shown. Repeated phyla/genera names omitted for easier visualisation. Full taxonomic assignment, indicator and significance values of all bacterial OTUs are reported in Supplementary Data S4.)*

**FIGURE 7:**
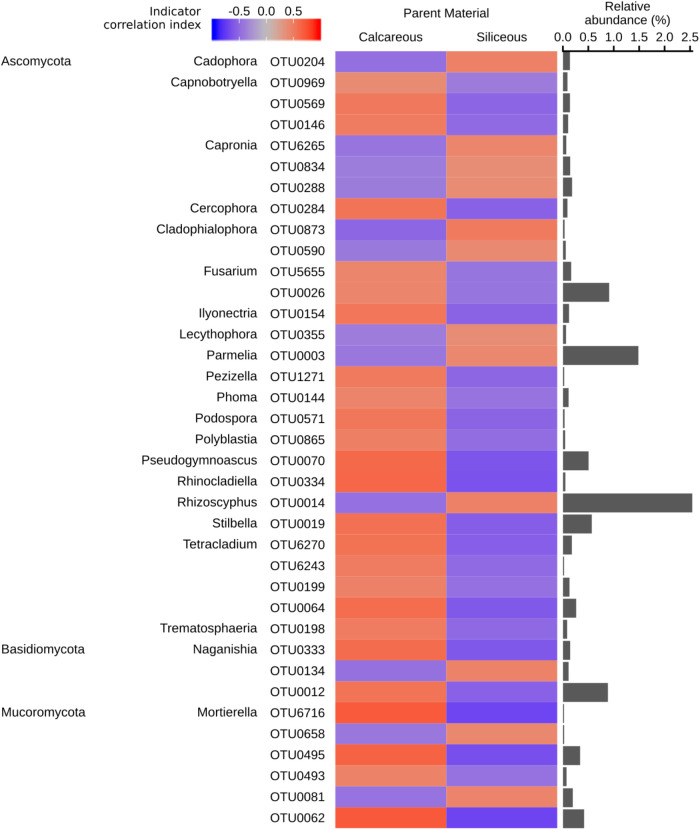
Fungal genera identified as significant indicators (*q* < 0.05) of calcareous or siliceous parent material (strength of correlation index > 0.4), grouped by phyla. The colour of the heatmap represents the strength of indication. The bars represent the relative abundance of each indicator OTU in all the samples. Indicator OTUs with a relative abundance of < 0.01% are not shown. Repeated phyla/genera names omitted for easier visualisation. Full taxonomic assignment, indicator and significance values of all fungal OTUs are reported in Supplementary Data S5.

As soil pH exhibited the strongest effect among soil parameters on bacterial α-diversity, as well as bacterial and fungal community structures, we decided to perform an indicator species analysis focussing on parent material as target category. From this analysis, we were able to identify 1,599 bacterial and 319 fungal OTUs, which were significantly (*q* < 0.05) associated with type of parent material and classifiable at the genus level. Bacterial and fungal OTUs assigned at different taxonomic levels are also reported in Supplementary Data S4, S5. Overall, the correlation indices of the bacterial OTUs to either type of parent material were significantly higher than those of the fungal OTUs, as shown by the colour strength of the respective heat maps in Figure 6, 7.

The majority of bacterial indicator OTUs for siliceous parent material belonged to the phylum *Acidobacteria* (e.g., the genus *Candidatus Solibacter*), with the notable exception of the genus *Blastocatella* (*Acidobacteria*), which exhibited a strong association with calcareous parent material (Figure 6). Furthermore, several bacterial indicator OTUs of the genus *Pirellula* and *Pir4 lineage* belonging to the phylum *Planctomycetes* were found to associate with calcareous soils. A highly abundant bacterial indicator OTU related to *Bradyrhizobium* (*Proteobacteria*) was characteristic for calcareous parent material. Several OTUs affiliated to the genus *Chthoniobacter* (phylum *Verrucomicrobia*) were associated with calcareous soils (Figure 6).

In general, the trends for fungal indicator OTUs were less clear. OTUs from the genus *Mortierella* belonging to the phylum *Mucoromycota* and the basidiomyceteous yeast of the genus *Naganishia* showed a mixed response, indicating no preference for either type of parent material. Ericoid mycorrhizal fungus *Rhizoscyphus* and lichenized fungus *Parmelia* were associated with siliceous soils (Figure 7).

## Discussion

While richness and compositional patterns along climatic gradients such as elevation have often been studied for plants (Gottfried et al., 2012; Pauli et al., 2012; Matteodo et al., 2013; Wipf et al., 2013; Rumpf et al., 2018; Steinbauer et al., 2018), only limited knowledge exists of bacterial and fungal diversity and community structure in high alpine soils. Here, we studied how the bacterial and fungal diversity and community structure changes with biotic, climatic and edaphic conditions on 10 GLORIA summits of the Swiss Alps, ranging from 2360 to 3212 m a.s.l. and differing in parent material (calcareous and siliceous). In line with other studies in the European Alps (Dullinger et al., 2003; Pauli et al., 2007), vegetation cover and species richness of vascular plants decreased with increasing elevation from the lowest summits, exhibiting lower-alpine grassland communities, to the highest, scarcely vegetated summits of the nival belt. Higher plant diversity and cover might be expected to cause a higher productivity of roots, which in turn results in a greater carbon input into soils from root necromass and root exudation (Mommer et al., 2016), thereby sustaining higher microbial biomass in soils at lower elevations. In line with this, soil DNA content, as a proxy for total biomass in soils, was lower on mountain summits at higher elevations, which mirrored the aforementioned trend of decreasing plant richness and cover with elevation. Soil DNA content was also closely related with SOC contents. A higher above and belowground productivity with decreasing elevation is associated with greater soil organic matter stocks in these poorly developed alpine soils, supporting the close link between above and belowground communities also over longer time scales.

### Microbial Diversities Are More Susceptible to Changes in Elevation Than Aspect

We hypothesised that with increasing elevation bacterial and fungal α-diversity will decline. Our results confirm this in the case of bacteria, where richness and Shannon diversity decreased with increasing elevation, which is in line with previous reports (Bryant et al., 2008; Li et al., 2018; and others). Fungal α-diversity, however, showed no significant change. This finding adds to several other studies showing no consistent relationship between elevation and fungal α-diversity (Nottingham et al., 2016; Tian et al., 2017; Ni et al., 2018).

In general, due to the varying effects of vegetation and soil properties along elevational gradients, it is difficult to examine the direct effect of elevation on microbial diversities. Thus, studies with more consistent key variables such as soil pH would be worthwhile to shed more light on the question. Furthermore, analysis of fungal guilds revealed no clear patterns of Shannon diversity of the different guilds with elevation apart from wood saprotrophs which exhibited a significant decrease in the region SN2. We therefore assume that the response of fungal α-diversity with elevation (temperature constraints) and upward shifts of plants (climate warming) is less pronounced than that of bacteria. Further studies are needed to improve our understanding of plant–microbial (above and belowground) interactions on mountain summits, such as shifts of diversity with climate change.

As shown by liner mixed-effects regression and PERMANOVA analyses, the effects we observed of aspect on microbial α-diversity and community structure were less clear. Only in interaction with elevation did aspect exhibit an influence on microbial α-diversity, and that only for bacteria. For example, west-exposed slopes had the strongest impact on α-diversity, which might be associated with higher soil temperature and/or shift in host plants. Furthermore, although the difference between mean soil temperatures of the coldest soils on northern aspects and the warmest soils on southern aspects within a single summit was as high as 4.5°C in the summer months, the impact of slope aspect on bacterial and fungal community structures was only marginal compared to that of summit. These findings suggest that summit, and as such elevation, are more important for shaping microbial community structures than aspect. This partially confirms our second hypothesis, however the influence of aspect is less than previously assumed. Interestingly, a study by Wu et al. (2017) found that bacterial community composition changed with aspect when comparing samples from sunnier versus shadier aspects of a mountain ridge (1500 m a.s.l.) in New Zealand. This study, however, has also shown that the influence of aspect appears to impact the bacterial community composition over smaller distances, yet elevation influences bacterial communities over larger distances. Unfortunately, fungal communities have not been analysed in in their study.

### Relationship Between Plant and Microbial Richness Is Elevation-Dependent

While numerous microbial surveys have been carried out on alpine soils, several contrasting patterns of microbial taxon richness along elevational gradients have been documented (Bryant et al., 2008; Singh et al., 2013, 2014; Zhang et al., 2015; Lanzén et al., 2016), suggesting that the co-varying effects of vegetation, soil properties, topography and climatic variables with elevation on microbial α-diversity cannot easily be separated. In our study, plant and microbial α-diversities did not appear related, similar to findings of Lanzén et al. (2015) and Prober et al. (2015). We did find, however, an elevation-dependent relationship between plant and microbial α-diversities. Our findings suggest that the shift in vegetation typology (bryophytes, lichen, vascular plants), as well as plant richness lead to the increase of microbial α-diversities, however this effect is apparent only with increasing elevation. In this respect, higher elevations are particularly important due to the pronounced differences in topoclimates toward the summits (Barry, 2008; Winkler et al., 2016). Moreover, the aforementioned studies by Lanzén et al. (2015) and Prober et al. (2015) were performed in grassland ecosystems, which exhibit a high pool of organic carbon available to soil microbes and plants, thus potentially masking interactions between them. However, at higher elevations the availability of SOC decreases, as we have also shown in this study, allowing us to detect a relationship between microbial α-diversities and plant richness. These findings were also shown by Porazinska et al. (2018), suggesting that in C-deficient soils there is a dependency of microbes on inputs from plants.

### Relative Abundance of Microbial Taxa Changes With Elevation

As elevation significantly influenced the microbial communities, we specifically examined its impact on the relative abundances of bacterial and fungal taxa. The most abundant bacterial phyla in our study were *Proteobacteria*, *Acidobacteria*, *Chloroflexi*, *Planctomycetes* and *Verrucomicrobia.* They include common representatives from copiotrophic groups (*Proteobacteria*) and taxa with mainly oligotrophic lifestyles (*Chloroflexi, Acidobacteria*, *Verrucomicroba*) according to the life strategy concept as described by Fierer et al. (2007). The distribution of the most abundant bacterial phyla was similar to that described in other alpine soils from the alpine-nival zone (Frey et al., 2016; Li et al., 2018; Ren et al., 2018). The strongest responses to a change in elevation were shown by bacteria of the phyla *Chloroflexi*, *Planctomycetes, Proteobacteria* (in particular those of *Alpha*, *Beta* and *Gammaproteobacteria*) and *Verrucomicrobia*.

*Chloroflexi* were on average abundant (13.7%) and highly diverse (1,106 OTUs), and their relative abundance significantly increased with elevation in all regions. This phylum was previously found to be abundant on a mountain ridge at ‘Muot da Barba Peider’ in the Swiss Alps (2960 m a.s.l.) both in the active layer as well in permafrost soils (Frey et al., 2016). Given the lack of ecological and physiological information on the various uncultured members of this phylum, it is currently difficult to speculate about their potential adaptation mechanisms on summits at higher elevations. *Chloroflexi* are a filamentous and metabolically highly diverse group (Hanada et al., 2002). Thus, different physiological strategies might be responsible for them coping with the harsh environments in soils at higher elevations (Costello and Schmidt, 2006). *Ktedonobacteria* OTUs (phylum *Chloroflexi*) mainly dominated summit soils at higher elevations, similar to findings for high elevational barren soils (Schmidt et al., 2018). Taxa within this class are reported to be prominent in cold environments (Barton et al., 2014; Kim et al., 2015; Tebo et al., 2015) and are known to have *Actinomycetes*-like morphology producing an array of secondary metabolites (Yabe et al., 2017). Overall, we suggest that these chemolithotrophic microbes are important for biogeochemical cycles in alpine summits and may decline in relative abundance with upward shifts of plants. Through the accumulation of plant litter on summits at high elevations these microbes might be outcompeted by more copiotrophic taxa. However, our understanding of their diversity and ecological significance is still very limited and further studies are needed, in particular with regards to the impact of global climate change on mountain summits.

Relative abundances of *Proteobacteria* decreased significantly with increasing elevation. Members of *Proteobacteria* are known to have copiotrophic lifestyles (Fierer et al., 2007), which may explain why these taxa were less abundant in the harsh and oligotrophic soil environments at higher elevations. The decline of *Planctomycetes* OTUs (e.g., with genus *Pirellula* and *Pir4* lineage) with increasing elevation is difficult to interpret. *Planctomycetes* are a widespread and numerically abundant microbial group in alpine (Grube et al., 2012; Frey et al., 2016) or in tundra soils (Ivanova et al., 2016). Their lifestyles are generally unknown. Members of this phylum can be lichen-associated (Grube et al., 2012) and were also identified as degraders of bacterial polysaccharides produced by other soil bacteria (Wang et al., 2015; Rime et al., 2016). Nevertheless, this phylum represents an interesting group due to its unusual features such as intracellular compartmentalisation and lack of peptidoglycan in their cell walls (Bödeker et al., 2017; Storesund et al., 2018), making it difficult to further speculate about its fate under climate warming.

Soil fungi have pivotal ecological roles as decomposers, pathogens and mycorrhizal symbionts. Alterations of their diversity arising from climate change could have substantial effects on ecosystem functions and plant establishment in soils at high elevation (Newsham et al., 2016). Fungi showed substantial variability in diversity and community composition between the different mountain summits, which is in line with a fungal compositional analysis in the Swiss National Park performed by Brunner et al. (2017). The dominance of *Ascomycota* (56.7%; 2,940 OTUs) and *Basidiomycota* (23.5%; 1,208 OTUs) reflects currently known fungal patterns in the Alps and the Arctic (Yao et al., 2013; Timling et al., 2014; Frey et al., 2016; Malard and Pearce, 2018; Ni et al., 2018). The fungal class that presented the highest richness in OTUs was that of *Agaricomycetes*. The dominance of them within fungal communities was also found in studies of the Tibetan Plateau (Yao et al., 2013; Wang et al., 2015). *Agaricomycetes* decreased with elevation, which is consistent with Tian et al. (2017) and could be due to the decreasing abundance of ectomycorrhizal host plants with increasing elevation.

The polyphyletic fungal group formerly known as *Zygomycota* (Hibbett et al., 2007), and more recently divided into the phyla *Mucoromycota* and *Zoopagomycota* (Spatafora et al., 2016), increased in abundance with elevation in all regions. In the case of *Mucoromycota* which are fast-growing soil-inhabiting saprotrophic fungi, mostly OTUs of the genus *Mortierella* were found to be more abundant at higher elevations. Fungal saprotrophs such as *Mortierella* and *Mucor* are known to be opportunistic alpine and subalpine “snow moulds” (Schmidt et al., 2012) that are likely able to colonise mountain summits at higher elevations due to their tolerance to low temperatures (Frey et al., 2016).

### Soil pH Is the Dominant Driver for Shaping Microbial Community Structures

In the present study, the main driver of microbial community structures, although to a lesser extent for fungi, was soil pH (originating from the different parent material). This is in agreement with previous reports (Rousk et al., 2010; Lanzén et al., 2015) and also confirms our third hypothesis. In addition, soil pH was also a strong driver of bacterial α-diversity, resulting in the increase of bacterial richness and Shannon diversity with increasing pH. Numerous studies documented that soil pH is the strongest driver of bacterial communities (Fierer, 2017) while its impacts on fungi are thought to be weaker (Rousk et al., 2010) or even absent with regards to fungal richness (Coince et al. (2014). The strong influence of pH on bacterial communities is suggested to be due to the narrow pH ranges for optimal growth of bacteria, as opposed to the weaker influence on fungi which exhibit in general a wider pH tolerance (Rousk et al., 2010). The importance of parent material on composition of bacterial communities has already been reported for other cold ecosystems (Lazzaro et al., 2009; Larouche et al., 2012; Reith et al., 2015; Tytgat et al., 2016; Yashiro et al., 2016; Li et al., 2018). Parent material and soil pH are closely related since bedrock, soil age (years of weathering) and plant cover influence soil pH (Lazzaro et al., 2009). Soil pH is an excellent integrator of soil nutrient availability since differences in hydrogen ion concentrations affect the capacity to hold charged ions in soils (Glassman et al., 2017). Besides soil pH, we found other factors (e.g., SOC and C:N ratio, soil temperature) to play weak, yet significant roles for microbial diversity and community structure, which is in line with previous studies of alpine environments (Geml et al., 2014; Lanzén et al., 2015; Ren et al., 2018).

### Microbial Indicators for Parent Material

We further explored which taxa drove the observed shifts in β-diversity as a result of the two types of bedrock originating from different parent material. *Acidobacteria* OTUs are commonly found in soils with low pH (Jones et al., 2009). Here, we found several OTUs of this phylum, however, with a mixed response to soil pH driven by the parent material. For example, *Blastocatella* OTUs were associated with calcareous soils (higher soil pH) and *Candidatus Solibacter* OTUs with siliceous soils (lower soil pH). Variation of *Acidobacteria* abundance along soil pH has also been shown previously (Männistö et al., 2007; Mukherjee et al., 2014; Yuan et al., 2017).

We found several OTUs of the genus *Chthoniobacter* (phylum *Verrucomicrobia*), which were indicators for calcareous bedrock. Members of this genus were within the 10 most abundant OTUs in our soils and are known to be involved in the transformation of organic carbon compounds in soil (Kant et al., 2011). *Chthoniobacter* was also found in the core microbiome of permafrost samples in the Tibetan Plateau (Guan et al., 2013) and other mountain soils (Rime et al., 2015; Wu et al., 2017; Shao and Gao, 2018). We suggest that these taxa are both well-adapted to cold environments and important members of mountain ecosystems in the Swiss Alps. The most abundant OTUs found in our study were from the genus *Bradyrhizobium* (class *Alphaproteobacteria*). They were strongly associated with summits on siliceous bedrock. Members of *Bradyrhizobiaceae* form nitrogen-fixing root-nodules with legumes while their predominance in mountain summits has not been reported so far.

Most of the fungal indicators showed a much weaker association to the type of parent material than bacteria indicating that soil pH was a stronger predictor for bacterial community structures than fungal ones. Fungal indicator OTUs were dominated by endophytes/dark septate endophytes (e.g., *Rhizoscyphus*), by saprotrophs (e.g., *Tetracladium*, *Naganishia* and *Mortierella*) or by a lichenized (e.g., *Parmelia*) fungi. The dominant fungal endophyte *Rhizoscyphus*, among the most abundant OTUs found in our soils, was associated with siliceous parent material. A previous study reported that *Rhizoscyphus* sp. show contrasting preferences for ridge or snow bed soils of high-alpine sites (Yao et al., 2013). Some members of the genus *Rhizoscyphus* are known to be associated with bryophytes (Timling et al., 2014), others are root endophytes (mycorrhizal) of ericaceous plants (Zijlstra et al., 2005), the latter occurring commonly in acidic soils. In contrast, OTUs of *Tetracladium* within the order *Heliotales* were indicators for calcareous soils. *Tetracladium*, a hyphomycete fungi can often be found on plant debris, indicating that this genus plays a role in litter degradation (Klaubauf et al., 2010). *Tetracladium* was also the dominant genus found in soil and lake sediments of maritime in Antarctica (Bridge and Newsham, 2009; Tsuji et al., 2013). These habitats have, in general, a high soil pH (around 8.0), which may explain the occurrence of *Tetracladium* OTUs in calcareous soils of mountain summits. Furthermore, several OTUs of saprotrophous *Mortierella* and *Naganishia*, which are among the 10 most abundant fungal genera, were indicators for both siliceous and calcareous soils, which explains their ubiquitous distribution and high abundance on mountain summits.

Overall, all these genera with their saprotrophic lifestyles undergo a selection in harsh environments and play several important ecological roles in our mountain summit soils, yet further investigations are needed to understand the association of different fungal OTUs to particular parent materials of alpine soils.

## Conclusion

Our study was the first to examine the soil microbiome of GLORIA mountain summits in the Swiss Alps. The summit soils were found to contain highly diverse microbial communities as a result of highly variable local environmental conditions among the summits, such as differences in parent material, soil properties, climatic variables and vegetation cover.

As hypothesised, bacterial α-diversity was observed to decline with increasing elevation (both alone and in interaction with other environmental variables), however soil pH proved to be its strongest driver. Fungal α-diversity, in contrast, was not significantly influenced by pH or elevation alone, instead vegetation in interaction with elevation was the strongest predictor, indicating a close relationship between vegetation and fungi particularly at higher elevations.

Despite its known effect on temperature and other soil properties, slope aspect had only a marginal impact on microbial community structure. Instead, soil pH, was also the strongest driver structuring bacterial communities as well as fungal communities, however to a lesser extent. These shifts in microbial β-diversity were further confirmed by the differing responses of bacterial and fungal taxa to soil pH and elevation. This study illustrates that soil pH is the most important predictor for shaping microbial communities in mountain summit soils of the European Alps. In order to elucidate the effects of other parameters on the soil microbiome of mountain summits in more detail, further investigations with comparable soil pH would be worthwhile.

As we have seen, bacterial communities tend to be sensitive to environmental perturbations and may respond strongly to a warming climate. Fungi, however, seem overall to respond weakly to environmental changes and thus possibly also to warming. However, due to the heterogeneity of the sampled microbial communities, in particular those of fungi, as seen by the differing responses of individual taxa to elevation, such generalised statements may mask underlying trends and therefore should be interpreted with care.

This study represents an important first characterisation of mountain summit soils and paves the ground for further characterisation of microbial communities within mountain ecosystems, and the related global carbon and nutrient cycles. Due to the strong correlation between elevation and temperature in alpine regions, our findings relating to elevation gradients may be extrapolated to predict the future response of microbial communities to global climate change. In particular, the multiple linear regression models presented here should serve as a useful baseline to estimate the extent to which such climatic changes influence the microbiota in soils of the European Alps.

## Author Contributions

BF, FH, CR, and SW designed the study. FH, CR, PV, and SW participated in sample collection. JD, AF, BF, FH, CR, J-PT, PV, and SW contributed to data collection. MA, JD, and BF performed data analysis. MA and BF wrote the manuscript. FH and SW contributed to the writing.

## Conflict of Interest Statement

The authors declare that the research was conducted in the absence of any commercial or financial relationships that could be construed as a potential conflict of interest.

## Footnotes

1
www.meteoswiss.admin.ch
