# Supplementary material for: The Soil Microbiome of GLORIA Mountain Summits in the Swiss Alps
Source: Front Microbiol. 2019 May 15;10:1080. doi: 10.3389/fmicb.2019.01080 (PMC6529532; doi:10.3389/fmicb.2019.01080)
Supplement: Supplementary file 6 [file Data_Sheet_6.PDF]

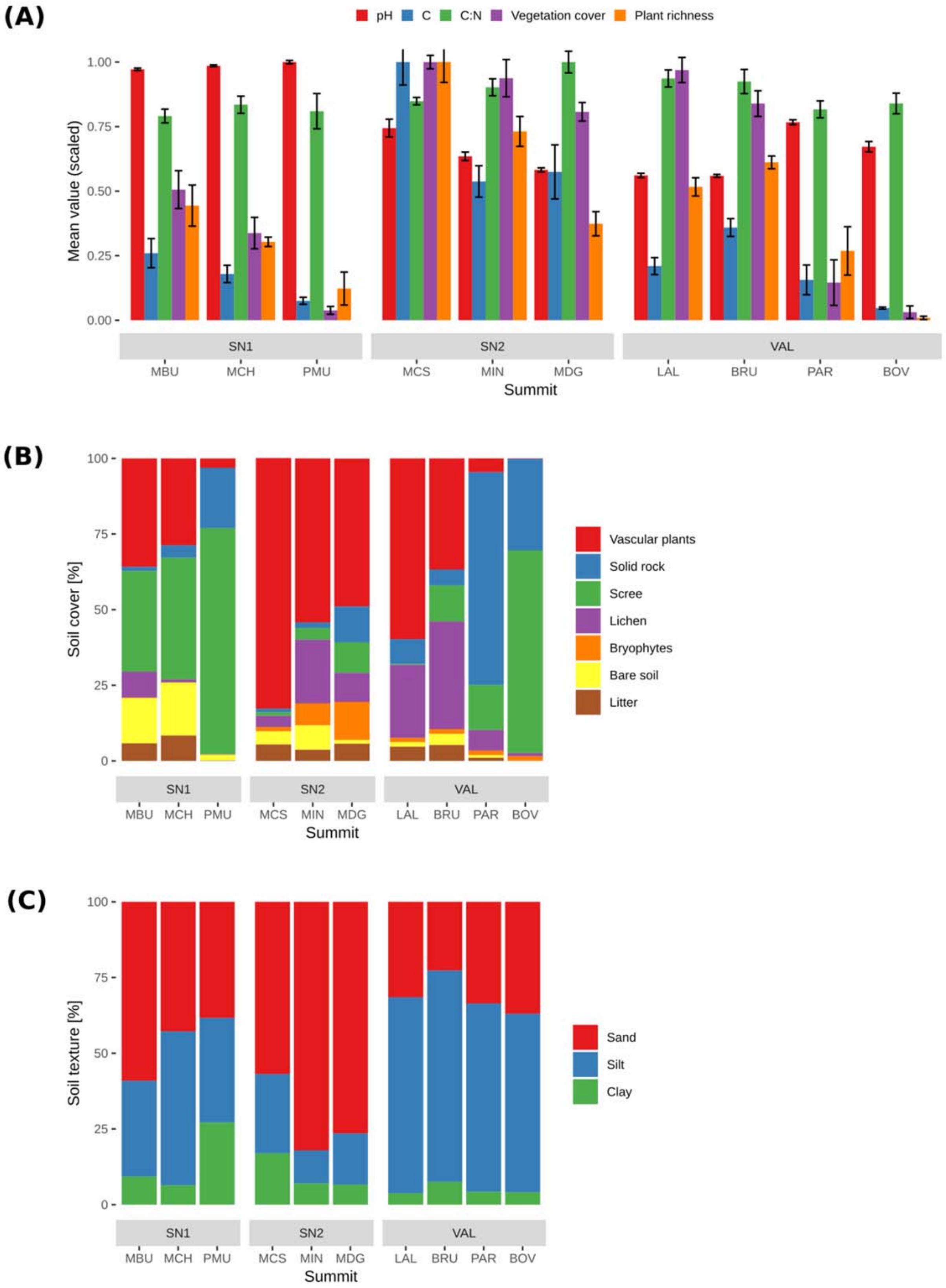

Figure S1. (A) Scaled bar plot of selected abiotic and biotic variables showing relative changes across summits (error bar shows standard error). Stacked bar plots of changes in (B) soil cover and (C) soil texture across summits. In all plots, summits are grouped by region and ordered by elevation (left – right) within. Region abbreviations: SN1 = Swiss National Park, calcareous parent material; SN2 = Swiss National Park, siliceous parent material; VAL = Valais, siliceous parent material.Summit abbreviations: MBU = Munt Buffalora; MCH = Munt Chavagl; PMU = Piz Murter; MCS = Mot sper Chamana Sesvenna; MIN = Minschuns; MDG = Mot dal Gajer; LAL = La Ly; BRU = Mont Brulé; PAR = Pointe du Parc; BOV = Pointe de Boveire.

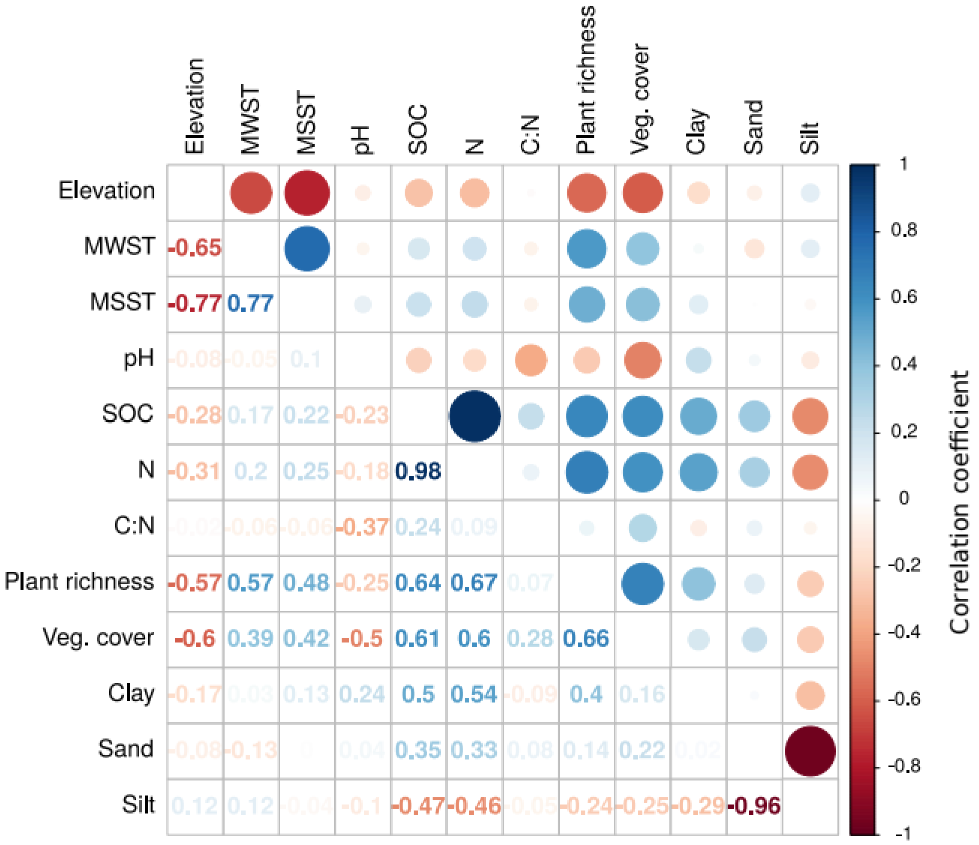

Figure S2. Correlation matrix of elevation and environmental parameters. Shown is the Pearson correlation coefficient. Abbreviations: MWST, mean winter soil temperature; MSST, mean summer soil temperature; SOC, soil organic carbon; N, nitrogen; C:N, carbon to nitrogen ratio; veg. cover, vegetation cover.

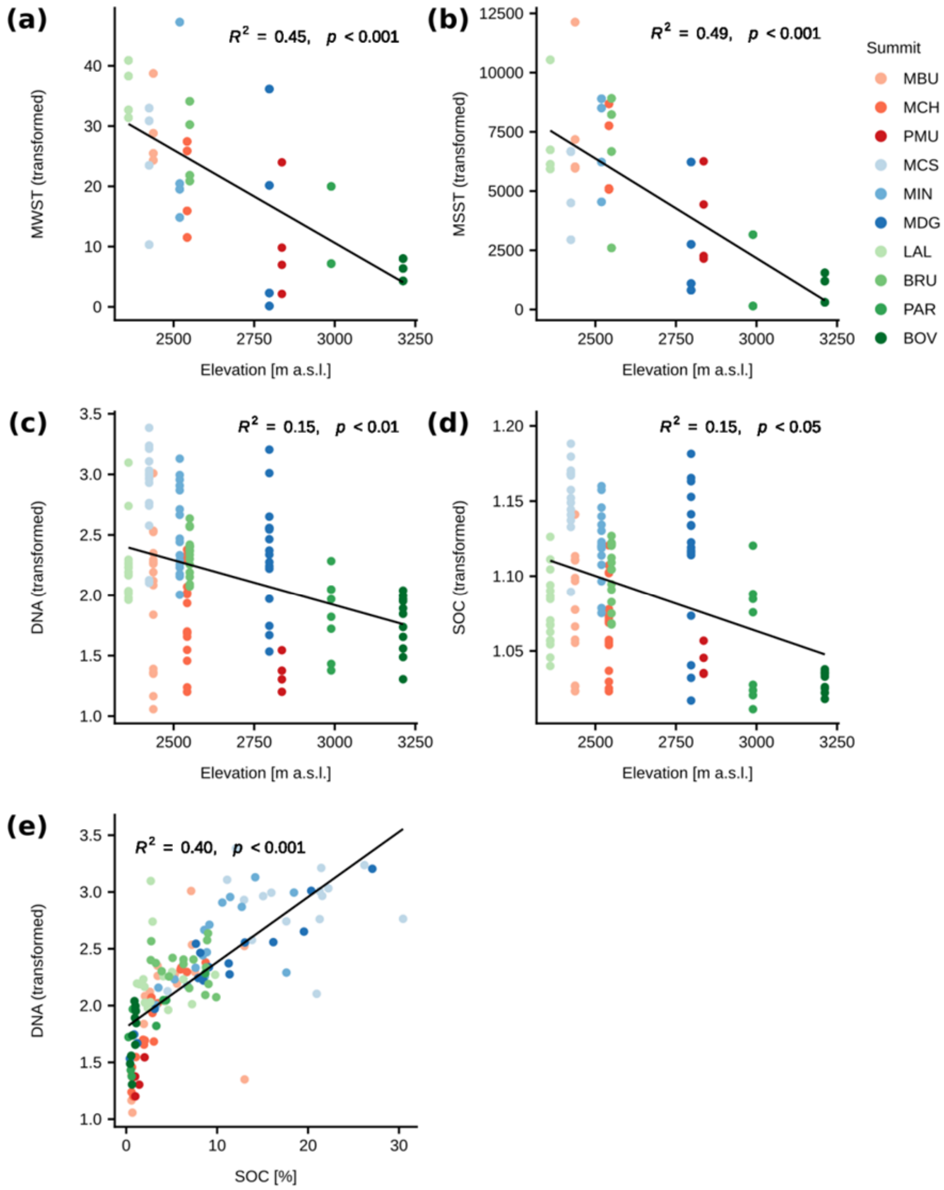

Figure S3. Relationships of seasonal soil temperatures during (a) winter and (b) summer, (c) soil DNA content and (d) SOC with elevation. (e) Relationship between soil DNA content and SOC. Regression lines were fitted using linear mixed-effects models (nested effects: region/summit/aspect; for temperature only: region/site, due to a single logger at each aspect). All response variables were transformed using Tukey's ladder of powers. Abbreviations: MWST, mean winter soil temperature; MSST, mean summer soil temperature; SOC, soil organic carbon. Summit abbreviations: MBU = Munt Buffalora; MCH = Munt Chavagl; PMU = Piz Murter; MCS = Mot sper Chamana Sesvenna; MIN = Minschuns; MDG = Mot dal Gajer; LAL = La Ly; BRU = Mont Brulé; PAR = Pointe du Parc; BOV = Pointe de Boveire. Colours correspond to regions: Red = SN1, Swiss National Park, calcareous parent material; Blue = SN2, Swiss National Park, siliceous parent material; Green = VAL, Valais, siliceous parent material.

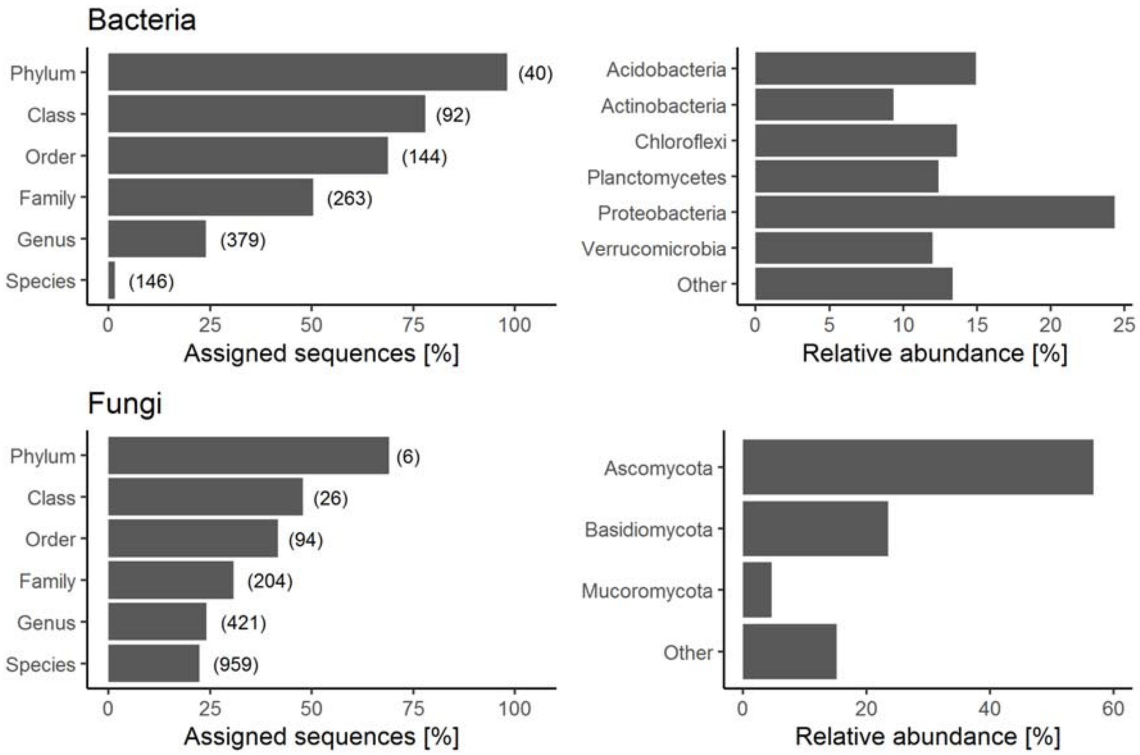

Figure S4. Assigned sequences for both bacteria and fungi for each taxonomic rank and their most abundant phyla.

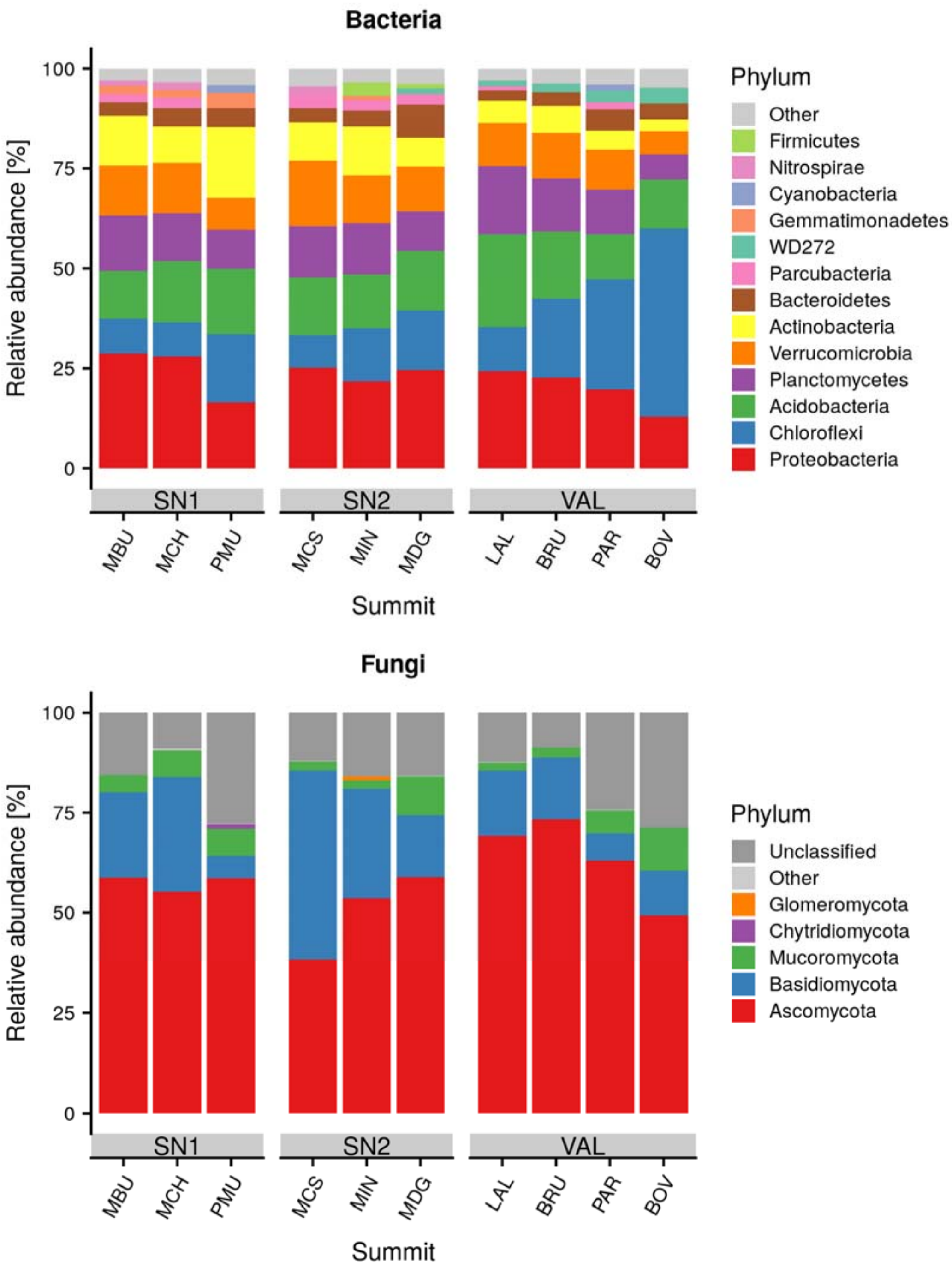

Figure S5. Relative abundances of bacterial and fungal most abundant phyla (> 1%) across different summits. “Other” represents all phyla with relative abundances < 1%. Summits are grouped by regions and ordered by increasing elevation (left – right) within. Region abbreviations: SN1 = Swiss National Park, calcareous parent material; SN2 = Swiss National Park, siliceous parent material; VAL = Valais, siliceous parent material. Summit abbreviations: MBU = Munt Buffalora; MCH = Munt Chavagl; PMU = Piz Murter; MCS = Mot sper Chamana Sesvenna; MIN = Minschuns; MDG = Mot dal Gajer; LAL = La Ly; BRU = Mont Brulé; PAR = Pointe du Parc; BOV = Pointe de Boveire.

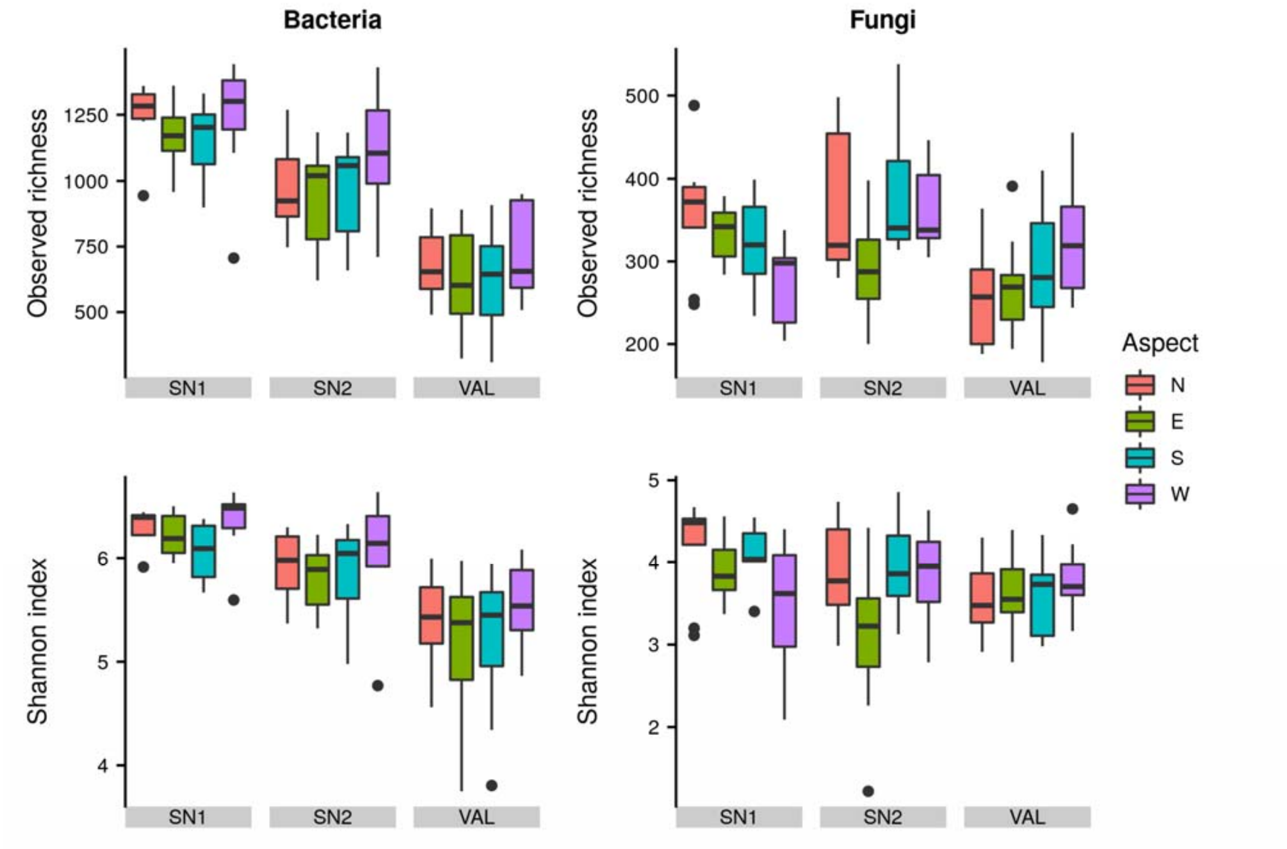

Figure S6. Variation of α-diversity (observed richness and Shannon index) of bacterial and fungal communities at different aspects, grouped by regions. Aspect abbreviations: N, north; E, east; S, south; W, west. Region abbreviations: SN1 = Swiss National Park, calcareous parent material; SN2 = Swiss National Park, siliceous parent material; VAL = Valais, siliceous parent material.

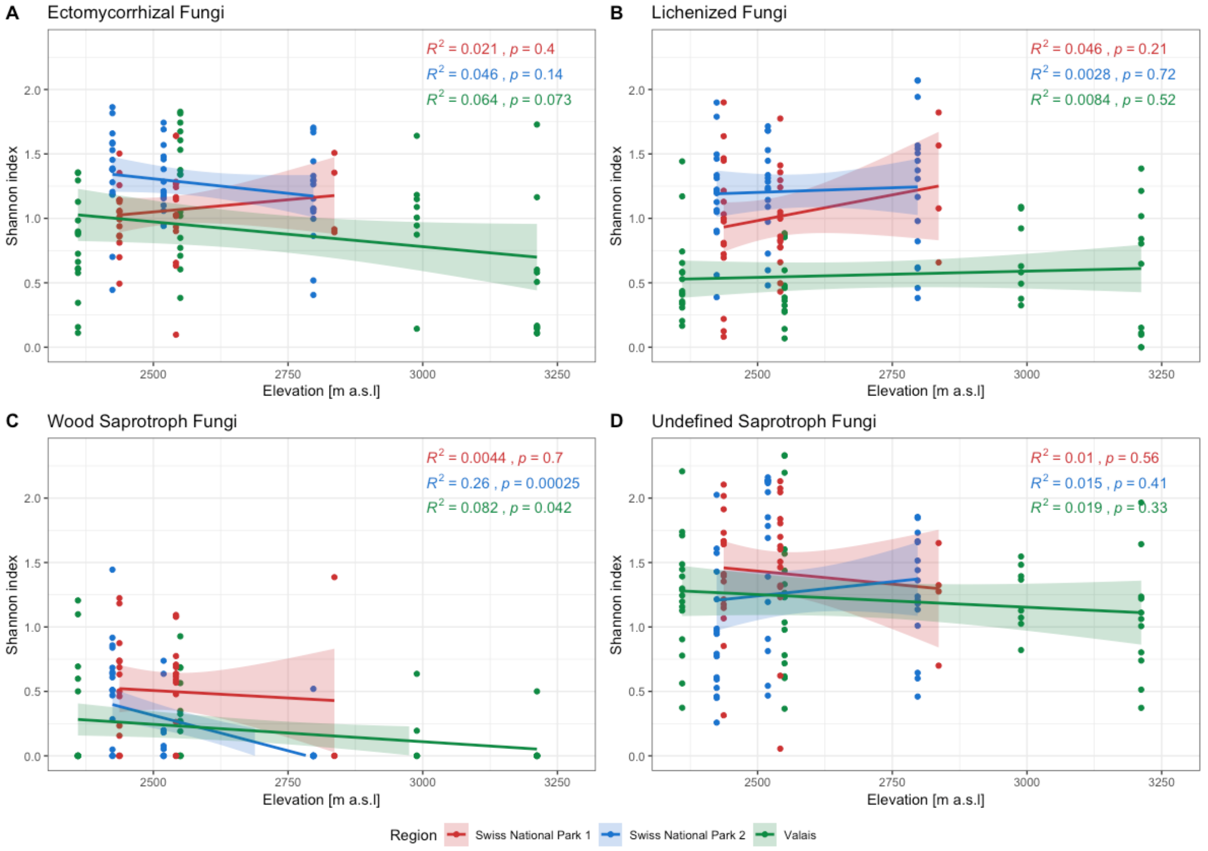

Figure S7. Pattern of Shannon diversity of the different fungal guilds with elevation. Fungal functional guilds were analysed by FUNGuild showing the four most abundant guilds, namely Ectomycorrhizal fungi, Lichenized fungi, Undefined saprotrophs, and Wood saprotrophs. Only the guild assignment with "highly probable" confidence rankings was accepted.
