# Supplementary material for: The Soil Microbiome of GLORIA Mountain Summits in the Swiss Alps
Source: Front Microbiol. 2019 May 15;10:1080. doi: 10.3389/fmicb.2019.01080 (PMC6529532; doi:10.3389/fmicb.2019.01080)
Supplement: Supplementary file 7 [file Data_Sheet_7.PDF]

Table S1. Relationships of abiotic and biotic variables with elevation using linear mixed-effects regression analysis.

**Environmental variables**	**Elevation F-value**
MWST [°C]	114.7 ***
MSST [°C]	52.7 ***
Soil pH	1.6 n.s.
SOC [%]	11.6 *
TN [%]	8.0*
C:N	0.5 n.s.
Soil Sand [%]	0.1 n.s.
Soil Clay [%]	0.2 n.s.
Soil Silt [%]	0.5 n.s.
Plant richness	40.4 ***
Vegetation cover [%]	71.1 ***
DNA [µg per weight (g) dry soil]	20.4 **

Shown is the F-statistic of the ANOVA output of linear mixed-effects models (nested effects: region/summit/aspect; for temperature only: region/summit, due to a single logger at each aspect). Environmental variables were transformed using Tukey's ladder of powers. Abbreviations: MWST, mean winter soil temperature; MSST, mean summer soil temperature; SOC, soil organic carbon; TN, total nitrogen; C:N, carbon to nitrogen ratio. Significance levels: n.s. (not significant), * $p < 0.05$, ** $p < 0.01$, *** $p < 0.001$.

Table S2. Effects of selected individual environmental variables and their interactions with elevation on bacterial α-diversity using linear mixed-effects regression analysis.

			S_{obs}			*Shannon*		
**Fixed effect**	**DF_{num}**	**DF_{den}**	**Norm. slope**	**F**	**R^2 marginal**	**Norm. slope**	**F**	**R^2 marginal**
Elevation	1	6	**-0.34**	**7.23 ***	**14.8%**	**-0.36**	**10.62 ***	**15.2%**
Aspect	3	24	-	2.11 n.s.	1.8%	-	2.68 n.s.	3.7%
Elevation × Aspect	4	23	-	**4.87 ****	**16.6%**	-	**5.04 ****	**17.8%**
MSST	1	26	-0.09	1.11 n.s.	0.8%	-012	1.28 n.s.	1.4%
MSST × Elevation	1	26	-0.11	2.18 n.s.	1.7%	-0.01	0.02 n.s.	0%
MWST	1	26	-0.11	2.64 n.s.	1.4%	-012	1.99 n.s.	1.6%
MWST × Elevation	1	26	-0.06	0.44 n.s.	0.4%	0.01	0.00 n.s.	0.0%
Soil pH	1	97	**0.48**	**16.70 *****	**26.4%**	**0.52**	**14.14 *****	**28.4%**
Soil pH × Elevation	1	97	-012	0.88 n.s.	0.08	-0.07	0.23 n.s.	0.0%
SOC	1	97	0.02	0.06 n.s.	0%	0.03	0.13 n.s.	0.1%
SOC × Elevation	1	97	**0.17**	**5.00 ***	**3.1 %**	**0.21**	**4.50***	**4.1%**
TN	1	97	-0.02	0.07 n.s.	0%	0.00	0.00 n.s.	0.0%
TN × Elevation	1	97	**0.17**	**4.03 ***	**2.8%**	0.20	3.63 n.s.	3.7%
C:N	1	97	0.05	1.36 n.s.	0.2%	0.09	2.59 n.s.	0.8%
C:N × Elevation	1	97	-0.02	0.11 n.s.	0%	0.01	0.03 n.s.	0%
Sand	1	97	0.12	0.82 n.s.	1.7%	0.31	3.58 n.s.	9.7%
Sand × Elevation	1	97	0.16	2.10 n.s.	2.0%	0.21	2.41 n.s.	3.2%
Silt	1	97	-0.03	0.04 n.s.	0.1%	-022	1.36 n.s.	5.3%
Silt × Elevation	1	97	-018	1.83 n.s.	2.9%	-0.22	2.20 n.s.	4.2%
Clay	1	97	-0.11	2.21 n.s.	1.2%	**-0.18**	**3.98 ***	**2.9%**
Clay × Elevation	1	97	-0.07	0.67 n.s.	0.5%	-0.05	0.19 n.s.	0.0%
Plant richness	1	97	-0.08	1.04 n.s.	0.6%	-0.10	1.13 n.s.	1.0%
Plant richness × Elevation	1	97	**0.23**	**7.05 ****	**7.7%**	**0.29**	**8.79 ****	**11.7%**
Vegetation cover	1	97	-0.09	1.42 n.s.	0.9%	-019	3.81 n.s.	3.4%
Vegetation cover × Elevation	1	97	**0.18**	**4.69 ***	**6.2%**	**0.22**	**5.81 ***	**9.4%**

Shown is the F-statistic of the ANOVA output of linear mixed-effects models (nested effects: region/summit/aspect). R^2 marginal indicates the proportion of variance explained by the fixed effects. Slope of regression line is normalised to account for differing units. Significant variables are in bold. Abbreviations: S_{obs}, observed richness; Shannon, Shannon diversity index; MSST, mean summer soil temperature; MWST, mean winter soil temperature; SOC, soil organic carbon; TN, total nitrogen; C:N, carbon to nitrogen ratio; DF, degrees of freedom; num, numerator; den, denominator. Shannon diversity index was transformed using Tukey's ladder of powers. Significance levels: n.s. (not significant), * $p < 0.05$, ** $p < 0.01$, *** $p < 0.001$.

Table S3. Effects of selected, individual environmental variables and their interactions with elevation on fungal α-diversity using linear mixed-effects regression analysis.

			S_{obs}			***Shannon***		
**Fixed effect**	**DF_{num}**	**DF_{den}**	**Norm. slope**	**F**	**R^2 marginal**	**Norm. slope**	**F**	**R^2 marginal**
Elevation	1	6	-0.14	1.25 n.s.	1.9%	-0.12	1.11 n.s.	1.5%
Aspect	3	24	-	1.06 n.s.	3.0%	-	1.39 n.s.	4.5%
Elevation × Aspect	4	23	-	0.47 n.s.	2.6%	-	0.71 n.s.	3.6%
MSST	1	26	0.07	0.33 n.s.	0.4%	0.08	0.49 n.s.	0.7%
MSST × Elevation	1	26	**0.19**	**5.23 ***	**6.7%**	**0.18**	**5.02 ***	**5.9%**
MWST	1	26	0.01	0.01 n.s.	0.0%	0.06	0.25 n.s.	0.3%
MWST × Elevation	1	26	**0.31**	**9.38 ****	**11.0%**	**0.24**	**5.50 ***	**6.3%**
Soil pH	1	97	-0.08	0.21 n.s.	0.6%	0.15	1.94 n.s.	2.3%
Soil pH × Elevation	1	97	0.02	0.01 ns.	0.0%	-0.13	0.82 n.s.	0.1%
SOC	1	97	0.20	3.67 n.s.	4.3%	0.0	0.00 n.s.	0.0%
SOC × Elevation	1	97	0.13	1.60 n.s.	1.6%	**0.26**	**6.12 ***	**5.9%**
TN	1	97	**0.24**	**4.66 ***	**5.8%**	0.01	0.01 n.s.	0.0%
TN × Elevation	1	97	0.11	1.02 n.s.	1.0%	**0.24**	**5.00 ***	**5.1%**
C:N	1	97	-0.03	0.14 n.s.	0.1%	-0.09	1.20 n.s.	0.9%
C:N × Elevation	1	97	**0.19**	**4.95 ***	**3.4%**	**0.27**	**8.79 ****	**6.4%**
Sand	1	97	**0.27**	**4.55 ***	**7.8%**	0.05	0.15 n.s.	0.2%
Sand × Elevation	1	97	0.19	2.18 n.s.	2.6%	**0.27**	**4.9 ***	**5.3%**
Silt	1	97	**-0.34**	**7.29 ****	**12.0%**	-0.09	0.53 n.s.	0.7%
Silt × Elevation	1	97	-0.12	1.02 n.s.	1.3%	**-0.25**	**4.58 ***	**5.1%**
Clay	1	97	0.11	0.99 n.s.	1.2%	0.11	1.19 n.s.	1.3%
Clay × Elevation	1	97	-012	1.19 n.s.	1.4%	0.04	0.18 n.s.	0.2%
Plant richness	1	97	0.07	0.48 n.s.	0.6%	-0.09	0.67 n.s.	0.8%
Plant richness × Elevation	1	97	**0.26**	**8.34 ****	**8.5%**	**0.33**	**14.46 *****	**13.2%**
Vegetation cover	1	97	0.07	0.40 n.s.	0.5%	-0.01	0.00 n.s.	0.0%
Vegetation cover × Elevation	1	97	**0.26**	**9.02 ****	**10.5%**	**0.26**	**10.04 ****	**9.7%**

Shown is the F-statistic of the ANOVA output of linear mixed-effects models (nested effects: region/summit/aspect). R^2 marginal indicates the proportion of variance explained by the fixed effects. Slope of regression line is normalised to account for differing units. Significant variables are in bold. Abbreviations: S_{obs}, observed richness; Shannon, Shannon diversity index; MSST, mean summer soil temperature; MWST, mean winter soil temperature; SOC, soil organic carbon; TN, total nitrogen; C:N, carbon to nitrogen ratio; DF, degrees of freedom; num, numerator; den, denominator. Both observed richness and Shannon diversity indices was transformed using Tukey's ladder of powers. Significance levels: n.s. (not significant), * $p < 0.05$, ** $p < 0.01$, *** $p < 0.001$.
