# Supplementary material for: The Soil Microbiome of GLORIA Mountain Summits in the Swiss Alps
Source: Front Microbiol. 2019 May 15;10:1080. doi: 10.3389/fmicb.2019.01080 (PMC6529532; doi:10.3389/fmicb.2019.01080)
Supplement: Supplementary file 8 [file Data_Sheet_8.PDF]

Supplemental Data 1.
All sample data including bacterial and fungal alpha diversities (observed richness and Shannon diversity), geographic, climatic and environmental (biotic and abiotic) parameters. Shown are average values for each aspect at each summit, including standard deviation.
Definitions:

- Mean winter soil temp.: Mean hourly temperature from the beginning of December to the end of February for the years 2016 and 2017.
- Mean summer soil temp.: Mean hourly temperature from the beginning of June to the end of August for the years 2016 and 2017.
- Min. recorded temp.: Lowest individual hourly temperature measured from the beginning of 2016 to the end of 2017.
- Max. recorded temp.: Highest individual hourly temperature measured from the beginning of 2016 to the end of 2017.
- Growing season: Number of days between snow melt and new snow, averaged for the years 2016 and 2017.

Supplemental Data 2.
List of all bacterial OTUs, taxonomic assignment and number of sequences (counts) for each OTU as well as their relative abundance.

Supplemental Data 3.
List of all fungal OTUs, taxonomic assignment and number of sequences (counts) for each OTU as well as their relative abundance.

Supplemental Data 4.
List of significant ($q > 0.05$) bacterial indicator OTUs for cancerous and siliceous parent material.

Supplemental Data 5.
List of significant ($q > 0.05$) fungal indicator OTUs for cancerous and siliceous parent material.
